# Suppressive stroma-immune prognostic signature impedes immunotherapy in ovarian cancer and can be reversed by PDGFRB inhibitors

**DOI:** 10.1186/s12967-023-04422-x

**Published:** 2023-09-01

**Authors:** Dong Yang, Mei-Han Duan, Qiu-Er Yuan, Zhi-Ling Li, Chuang-Hua Luo, Lan-Yue Cui, Li-Chao Li, Ying Xiao, Xian-Ying Zhu, Hai-Liang Zhang, Gong-Kan Feng, Guo-Chen Liu, Rong Deng, Jun-Dong Li, Xiao-Feng Zhu

**Affiliations:** 1 State Key Laboratory of Oncology in South China, Collaborative Innovation Center for Cancer Medicine, Guangdong Key Laboratory of Nasopharyngeal Carcinoma Diagnosis and Therapy, Sun Yat-Sen University Cancer Center, 651 Dongfeng Road East, Guangzhou, 510060 China; 2 Department of Gynecological Oncology, Sun Yat-Sen University Cancer Center, Guangzhou, China; 3 Department of Medical Imaging, Sun Yat-Sen University Cancer Center, Guangzhou, China; 4 Department of Intensive Care Unit, Sun Yat-Sen University Cancer Center, Guangzhou, China

**Keywords:** Ovarian cancer, Immunotherapy, Stroma, Prognostic signature

## Abstract

**Background:**

As the most lethal gynecologic cancer, ovarian cancer (OV) holds the potential of being immunotherapy-responsive. However, only modest therapeutic effects have been achieved by immunotherapies such as immune checkpoint blockade. This study aims to propose a generalized stroma-immune prognostic signature (SIPS) to identify OV patients who may benefit from immunotherapy.

**Methods:**

The 2097 OV patients included in the study were significant with high-grade serous ovarian cancer in the III/IV stage. The 470 immune-related signatures were collected and analyzed by the *Cox* regression and *Lasso* algorithm to generalize a credible SIPS. Correlations between the SIPS signature and tumor microenvironment were further analyzed. The critical immunosuppressive role of stroma indicated by the SIPS was further validated by targeting the major suppressive stroma component (CAFs, Cancer-associated fibroblasts) in vitro and in vivo. With four machine-learning methods predicting tumor immune subtypes, the stroma-immune signature was upgraded to a 23-gene signature.

**Results:**

The SIPS effectively discriminated the high-risk individuals in the training and validating cohorts, where the high SIPS succeeded in predicting worse survival in several immunotherapy cohorts. The SIPS signature was positively correlated with stroma components, especially CAFs and immunosuppressive cells in the tumor microenvironment, indicating the critical suppressive stroma-immune network. The combination of CAFs’ marker PDGFRB inhibitors and frontline PARP inhibitors substantially inhibited tumor growth and promoted the survival of OV-bearing mice. The stroma-immune signature was upgraded to a 23-gene signature to improve clinical utility. Several drug types that suppress stroma-immune signatures, such as EGFR inhibitors, could be candidates for potential immunotherapeutic combinations in ovarian cancer.

**Conclusions:**

The stroma-immune signature could efficiently predict the immunotherapeutic sensitivity of OV patients. Immunotherapy and auxiliary drugs targeting stroma could enhance immunotherapeutic efficacy in ovarian cancer.

**Supplementary Information:**

The online version contains supplementary material available at 10.1186/s12967-023-04422-x.

## Background

Ovarian cancer (OV), the deadliest gynecological cancer, has become the eighth leading cause of female cancer death [1]. Genomic instability, a critical factor that can significantly increase tumor mutation burden (TMB) and neoantigen production, especially “BRCAness,” can be detected in many OVs; thus, OV is recognized as immunogenic and potentially responsive to immunotherapy [2–5]. However, recent studies have reported that neither the immune monotherapy based on PD1/PDL1/CTLA4 blockade (objective response rate of 10 ~ 30%) nor the combination of immune checkpoint blockade and chemotherapy/PARP inhibition achieved satisfactory results [5–10].

The tumor microenvironment (TME), a cancer-cell-established variable ecosystem, is crucial in determining responsiveness and non-responsiveness to immunotherapies [11]. TME is classified into three subtypes: the hot subtype with inflamed T cells, the excluded subtype with stroma-confined T cells, and the cold subtype with absent T-cell infiltration [5]. According to the classification of OV (CLOVAR), OV is further classified into four distinct yet overlapping subtypes: immunoreactive, differentiated, proliferative, and mesenchymal subtype [5]. Based on pan-cancer analysis by Bagaev et al., OV was categorized as immune-enriched/fibrotic (IE/F), immune-enriched/non-fibrotic (IE), fibrotic, and desert subtype [12]. Accordingly, OV patients of hot, immunoreactive, or immune-enriched/non-fibrotic subtype had the best prognosis compared to other subtypes. Those inflamed tumor subtypes are characterized by anti-tumor immune cell infiltration, more potent activation of interferon signaling, higher level of chemokine secreting such as CXCL9/10, and less immune-suppressive stroma including CAFs, abnormal tumor endothelium, etc [5]. Therefore, identification and quantification of TME components could identify tumor subtypes and precisely discriminate patients responsive to immunotherapies.

Due to genomic and transcriptomic information, researchers recently aimed to screen potential prognostic biomarkers to model immune components in TME [13]. Several studies focused on the expression level of immune-related genes in tumor tissue. Ding et al. concluded the nine-gene signature containing UBD, GBP2, CXCL11, CXCL13, D4S234E, VSIG4, CXC3R1, C5AR1, and TFPI2 [14]. Shen et al. constructed a 129-gene immunogenic prognostic signature of which the majority were cytokine-related genes and antimicrobial-signaling-related genes [15]. Such signatures efficiently classify OV patients into high- and low-risk immune subgroups, where the high-risk subgroup tends to have more suppressive immune cells, such as M2 macrophages, and less positive immune cells, such as CD8^+^T cells, while the low-risk group is characterized by more positive immune cells and significant activation of interferon signaling and chemokine signaling [13]. However, few signatures in the previous studies emphasized the critical immunosuppressive role of stroma components in TME.

In the present study, to fully explore the immune status in the tumor microenvironment to find new biomarkers for the prediction of immunotherapeutic efficacy in OV patients and to provide new strategies for the treatment of the OV patients, a total of 470 immune-related signatures are submitted to develop the significant immune-related prognostic model in the 2097 OV patients from multiple cohorts. The correlation between the prognostic model and tumor microenvironment is further analyzed in the OV and other immunotherapeutic cohorts. Our work may shed light on the pathogenesis of OV tumors with immune resistance.

## Materials and methods

### Materials

Details of all inhibitors, primers’ sequences, and datasets mentioned in this research were displayed on GitHub (https://github.com/Yangd38).

### Data collection and processing

**Fig. 1:**
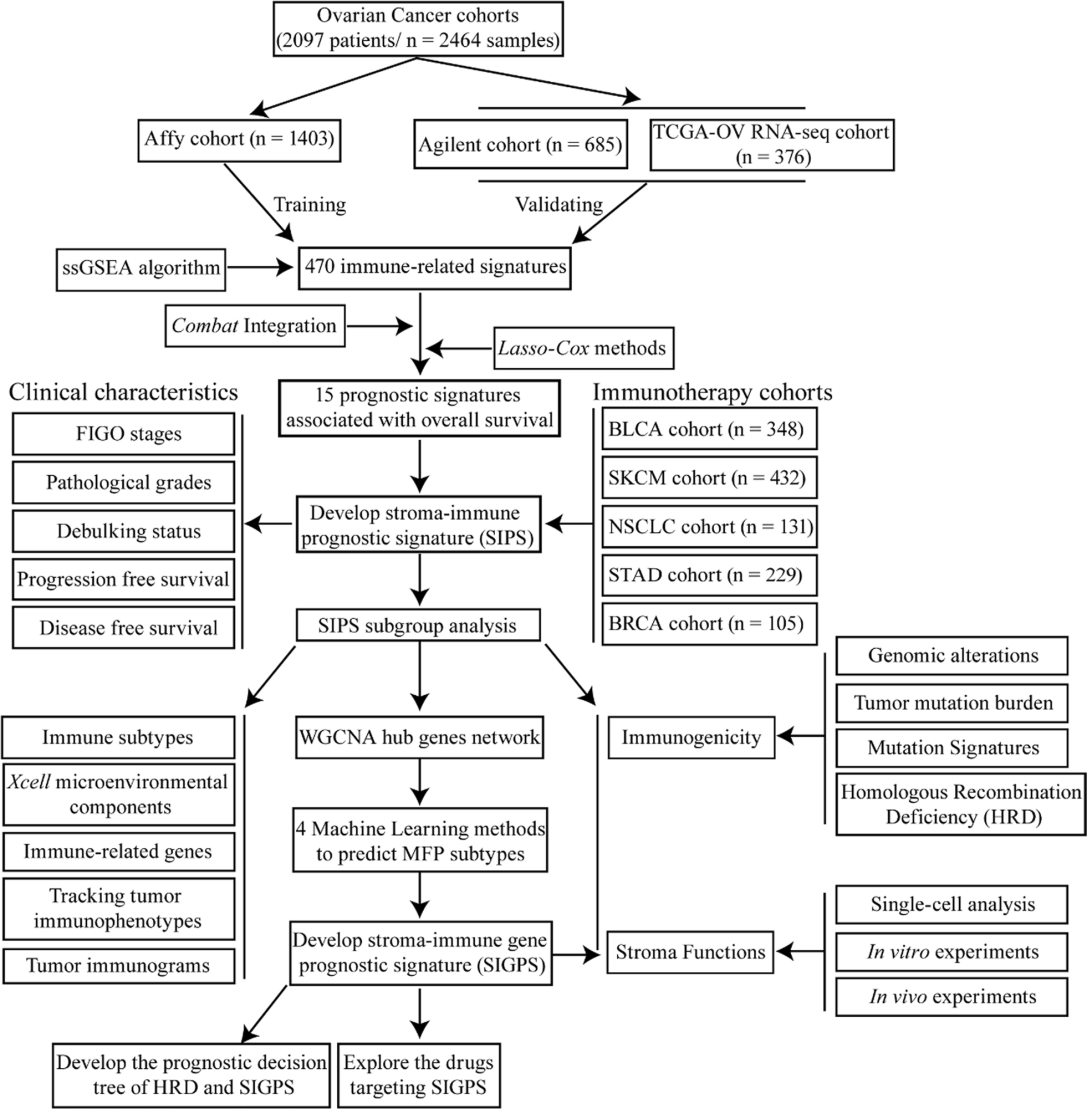
The study design and workflow

The RNA-seq data, SNV data (simple nucleotide variation), CNV data (copy number variation), and corresponding clinical information were obtained from the Cancer Genome Atlas cohorts (TCGA-OV). The microarray data of the Affy ovarian cancer cohort were extracted from Gene Expression Omnibus (GSE82191, GSE18520, GSE30161, GSE19829, GSE63885, GSE26193, GSE9891, GSE14764, GSE23554, GSE26712). The microarray data of the Agilent ovarian cancer cohort were extracted from Gene Expression Omnibus (GSE53963, GSE73614, GSE17260, GSE32062, GSE32063). The immunotherapeutic cohorts were collected from the five cancers, including Bladder Urothelial Carcinoma (BLCA), Skin Cutaneous Melanoma (SKCM), Non-small cell lung cancer (NSCLC), Gastrointestinal cancer (GC), Breast invasive carcinoma (BRCA). The single-cell sequencing data were obtained from Gene Expression Omnibus (GSE165897). The stroma-related datasets were also obtained from Gene Expression Omnibus (GSE115635, GSE38666, GSE9890, GSE164088). The corresponding clinical information of these immunotherapeutic cohorts was listed in Additional file 2: Supplementary Table S1. The drug-induced expression signatures were obtained from the CMAP database (https://clue.io/). The study design and workflow were presented in Fig. 1.

### Single sample Gene Set Enrichment Analysis (ssGSEA)

The 470 immune-related signatures (Additional file 3: Supplementary Table S2) were obtained from the work of Shiyuan et al. [16] and the MSigDB database [17]. The normalized enrichment scores (NESs) of 470 immune cell signatures in each ovarian cancer sample and the corresponding expression data were quantified by *ssGSEA* function in R package *GSVA* (v1.45.5) [18]. The potential batch effects between the NESs in different cohorts were removed by the *ComBat* function in R package *sva* (v3.44.0).

### Construction of the stroma-immune prognostic signature

The combated NESs of 470 immune-related signatures from the Affy cohort as the discovery cohort were analyzed by univariable Cox proportional hazards regression analysis. Twenty-two immune-related signatures were significantly correlated with overall survival (the adjusted *P*-value < 0.05, Additional file 4: Supplementary Table S3). Then, based on R package *glmnet* (v4.1-4), the *LASSO-Cox* regression model was applied to identify the most valuable prognostic factors among the 22 immune cell signatures in the discovery cohort. Ultimately, 15 immune-related signatures with nonzero coefficients were selected according to the minimized lambda. SIPS was constructed using the NESs of 15 immune cell signatures to multiply the regression coefficients derived from univariable Cox proportional hazards regression analysis. Then, SIPS was further averaged by the 15 signatures and normalized to 0 ~ 1 by subtracting the minimum and dividing by the range (maximum-minimum). Since overall survival records of patients in the BRCA cohort were unavailable, the parameters of SIPS in the Affy cohort were applied to calculate the SIPS of the patients from the BRCA cohort.

### The nomogram construction and ROC analysis

SIPS, FIGO stage, histopathological grade, and debulking status were used to establish a nomogram model to predict 3/5-year overall survival in the discovery and testing cohort based on R package *rms* (v6.3-0). The validation of the nomogram was processed by testing the discrimination and calibration abilities with the internal (discovery) and external (validation) sets, respectively. Moreover, the concordance index (C-index) and the Receiver Operating Characteristic (ROC) curves were also applied to evaluate the nomogram model. The ROC analysis was performed on R package *timeROC* (v0.4).

### Tumor microenvironment analysis

TME components were analyzed by *xCell* algorithm based on R package *immunedeconv* (v2.0.4). Default parameters were adopted in the R package *immunedeconv*. The tracking tumor immunophenotype (TIP) analysis was performed on the TIP website (http://biocc.hrbmu.edu.cn/TIP/index.jsp). The immunogram analysis was applied to explore the cancer-immunity interactions. The activation levels of 10 immunogram signatures were estimated based on *ssGSEA* function in R package *GSVA* (v1.45.5).

### Hub functional analysis

Differentiated expression of genes (DEGs) between the high and low SIPS subtypes in the Affy and TCGA-OV cohorts were identified by R package *Limma* (v3.52.4). RNA-seq data of the TCGA-OV cohort were collected to perform weighted gene co-expression network analysis (WGCNA) of the common differentiated genes between Affy and TCGA-OV cohort based on R package *WGCNA* (v1.71). Eigengenes from each module were collected to run the STRING protein–protein interaction (PPI) analysis and cluster eigengenes by k-means methods on the *STRING* Web (https://cn.string-db.org/) separately, out of which 60 most connected eigengenes in each module cluster were collected to run STRING PPI analysis. STRING PPI result was then analyzed to determine hub genes by *CytoHubba* (v0.1) function in *Cytoscape* (v3.9.1) to determine hub genes from the STRING PPI result. The R package *clusterProfiler* (v3.18.1) was applied for gene ontology (GO) biological process enrichment analysis of the hub genes. The results of the hub functional analysis were in the Additional file 5: Supplementary Table S4. Detail parameters were displayed in the corresponding Rscript file on Github (https://github.com/Yangd38).

### scRNA-seq data process and analysis

TME was analyzed based on 51,786 selected single cells from 11 HGSOC patients in GSE165897. R package *Seurat* (v4.2.0) was applied to run cell quality control and normalize the gene expression in each cell with default parameters. Each cell was defined by cell definition in this dataset, and cell clusters were displayed by uniform manifold approximation and projection (UMAP) algorithms. R package *scGSVA* (v0.0.11) was adopted to calculate each cell’s NESs of 15 immune-related signatures in the SIPS. The communication signaling network between tumor and stroma cells was examined by the R package *Cellchat* (v1.4.0).

### Genomic instability analysis

R package *scarHRD* (v0.0.1) was adopted to ratiocinate HRD Score, LOH (Loss of Heterozygosity), TAI (Telomeric Allelic Imbalance), LST (Large Scale Transitions) from the CNV data of TCGA-OV cohort. R package *maftools* (v2.12.0) was utilized to display the landscape of 30 most-mutated genes and calculate the TMB. R package *sigminer* (v2.1.8) was applied to determine the mutational signatures in each OV patient.

### Construction of 23-gene signature by machine-learning models

Four machine-learning models (*Boruta*, *xgboost*, *Random Forest*, *LASSO*) were applied to screen the generalized key genes to identify tumor immune-subtypes (MFP subtype). R package *Boruta* (v7.0.0), *xgboost* (v1.6.0.1), *caret* (v6.0-93) and *glmnet* (v4.1-4) were used to perform the machine-learning process.

### Decision tree modeling

The survival decision tree was built by R package *rpart* (v4.1.19), *partykit* (v1.2-16), and *survival* (v3.4-0). Overall survival and status of the TCGA-OV cohort were the response factors. HRD Score and SIGPS were independent variables, and other parameters adopted the default. The cutoff value of pruning was set to maximize the significance of the tree and simplify the tree.

### Connectivity map analysis

Potential drugs targeting stroma were selected using CMAP web (the largest perturbation-driven gene expression dataset) to screen drugs with expression-suppressing profiles similar to SIGPS genes. 49 common genes concluded by any three machine-learning methods were collected to search potential drugs on the CMAP web. Experimentally-examined L1000 sub-database was selected, and drugs with raw connective scores < 0 and FDR < 0.05 were selected. The results of CMAP analysis were in the Additional file 6: Supplementary Table S5.

### Cell culture

Ovarian cell lines SKOV3, OVCAR5, and TOV21G were purchased from ATCC. The ID8 cell line was a gift from Pro. Xia Xiaojun in Sun Yat-sen University Cancer Center. Trp53^−^Brca2^−^-ID8 cells were constructed by transfecting ID8 cells with lentiCRISPRv2-bsd-sgTp53 and lentiCRISPRv2-puro-sgBrca2 virus. Tp53^−^Brca2^−^ID8-Luc cells were built by transfecting Tp53^−^Brca2^−^ID8 cells with the PGF-GFP-LUC virus, and monoclonal cells were sorted out by flow cytometric sorting. ID8-OVA cells were constructed by transfecting ID8 cells with PCDH-puro-OVA. All cells were cultured in DMEM supplemented with 7% FBS and antibiotics (50 mg/mL penicillin/streptomycin). All cell lines were verified to be mycoplasma-free. The primary cancer-associated fibroblasts (CAFs) were isolated from an HGSOC patient’s tumor sample obtained from surgery in our cancer center. The detailed procedure of CAFs isolation and culture followed the protocol by Mercedes et al. [19]. Informed consent regarding the sample collection has signed by the patient, and all related procedures were performed with the approval of internal review and ethics boards in Sun Yat-sen University Cancer Center.

### RNA isolation and qRT-PCR

After treatment, total RNA from cell lines was isolated using EZ-press RNA Purification Kit (EZBioscience, cat: B0004D) and converted to cDNA using the HiScript II Q RT SuperMix for qPCR (Vazyme, cat: R223-01). qRT-PCR was performed according to the manufacturer’s protocol (ChamQ SYBR qPCR Master Mix (Vazyme, cat: Q311-02), Roche Applied Science LightCycler 480). The relative expressions of genes were calculated using the 2^−ΔΔ^Ct method, and GAPDH/β-actin was adopted as the control. The qPCR primers were in the Additional file 7: Supplementary Table S6.

### OT-I in vitro killing assay

Splenocytes isolated from OT-I mice were activated with 2 ng/ml OVA257–264 (N4, Sangon Biotech, cat: T510212) and 10 ng/ml IL-2 for 3 days. ID8 cells were pulsed by 1 ug/ml OVA peptide for 30 min. The activated CD8^+^T cells were co-cultured with ID8-OVA cells in RPMI 1640 medium supplemented with 2% FBS) at the ratios of 0.5:1 and 1:1 for 4 h, then cells were incubated with anti-CD45.1 and anti-caspase3 for 30 min, followed by flow cytometry to analyze the percentage of CD45^−^Caspase3^+^ cells.

### Animal experiments

The 6-week-old female C57BL/6 mice were purchased from the Guangdong GemPharmatech Co., Ltd (Guangzhou, China). All animal experiments were conducted following the institutional guidelines and approved by our cancer center’s Animal Care and Use Committee. C57BL/6j mice were intraperitoneally injected with 5 × 10^6^ Trp53^−/−^Brca2^−/−^-ID8-luc cells to construct animal models. Tumor-bearing mice were sorted by In Vivo Imaging System (IVIS; PerkinElmer, Inc.) after 3 weeks and randomly divided into four subgroups (Vehicle, Niraparib, Sunitinib, and Niraparib–Sunitinib subgroup; five mice per subgroup). Mice were treated with Niraparib 10 mg/kg and sunitinib 10 mg/kg intraperitoneal injection every 5 days. Tumor progression was monitored weekly by IVIS. The applied cytokines and chemical inhibitors were in the Additional file 8: Supplementary Table S7.

### Statistics

All values were presented as mean ± SEM. *Kruskal–Wallis* test and *Wilcoxon* test were adopted to compare differences among groups. R package *ggstatsplot* (v0.9.5) was used to plot the percentage stacked bar and analyze the statistical significance. *p < 0.05; **p < 0.01, ***p < 0.001 were defined to be statistically significant and* p* > 0.05 was *n.s.* (non-significant).

## Results

### Immune related signatures for the prognostic prediction of ovarian cancer

**Fig. 2:**
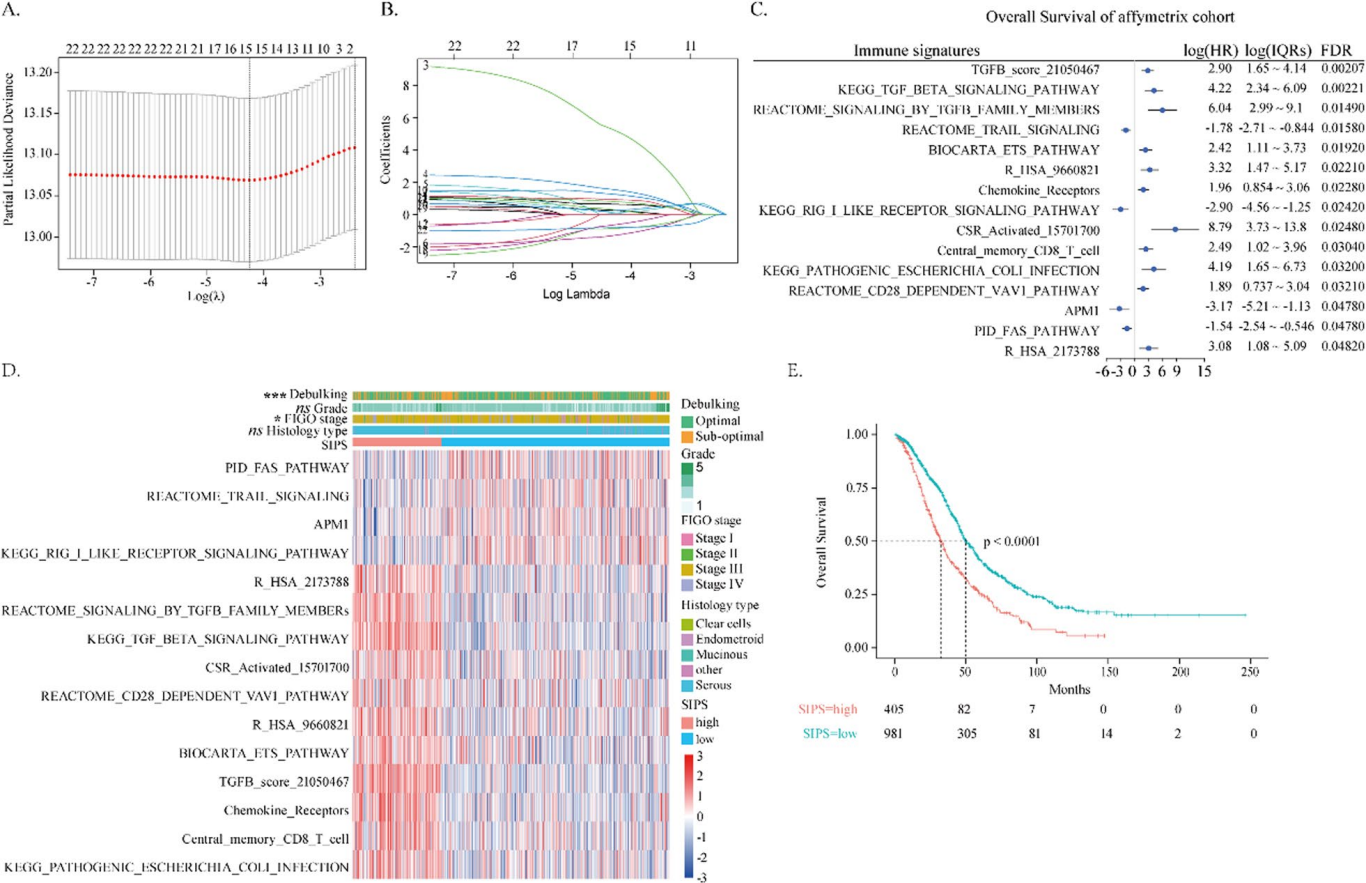
Immune related signatures for the prognostic prediction of ovarian cancer. **A** Partial likelihood revealed by the *LASSO* regression model in Affy cohort. **B** *LASSO* coefficient profiles of 15 selected immune-related signatures in Affy cohort. **A**, **B** The vertical dotted lines were drawn at optimal values by the minimum and 1-SE criteria. **C** The forest plot of the associations between the 15 immune-related signatures and overall survival in Affy cohort. The HR, log value of interquartile range (IQR), and false discovery rate (FDR) were determined by univariate Cox regression analysis. **D** The heatmap of Affy cohort plotted by the NESs of 15 immune-related signatures. **E** The Kaplan–Meier estimate of the overall survival in Affy cohort, divided by two SIPS subtypes

Three integrated cohorts (Affy, Agilent, and TCGA cohorts) containing 2097 ovarian cancer samples with overall survival information were used for prognostic model construction (Additional file 1: Fig. S1A–B). There were 15 out of 470 immune-related signatures significantly correlated with overall survival, which was selected by *LASSO-Cox* regression in the Affy cohort (Fig. 2A, B). The relationships between the 15 immune-related signatures and overall survival were illustrated in the forest plot (Fig. 2C). Previous studies have reported that the 15 immune-related signatures were correlated with chronic inflammation signatures and tumor stromal signatures [20, 21]. Thus, stroma-immune prognostic signature (SIPS) was constructed by integrating the 15 immune-related signatures. Patients in the Affy cohort were stratified into high and low SIPS subtypes according to the optimal cutoff of survival risk score (Fig. 2D). The Kaplan–Meier survival analysis indicated that patients with low SIPS had better overall survival, disease-free survival, and progress-free survival, compared to those with high SIPS (32.8 versus 50.0 months) (Fig. 2E, Additional file 1: Fig. S1C–D). To further examine the robustness of the SIPS model, SIPS performance was tested in the Agilent and TCGA-OV cohorts. Similarly, patients were stratified into high and low SIPS subtypes based on the corresponding optimal cutoff values (Additional file 1: Fig. S1E–F). Similar results were observed in the TCGA cohort and the Agilent cohort, where patients of the high SIPS subtype had significantly worse overall survival than those of the low SIPS subtype (40.4 versus 49.7 months, TCGA cohort) (51 versus 80 months, Agilent cohort) (Additional file 1: Fig. S1G–H).

To realize quantitative prediction of OV patient prognosis in the clinic, a nomogram that integrated both the SIPS and other clinical characteristics was constructed based on patients from the Affy cohort, the TCGA cohort, and the Agilent cohort, by which scores could quantify an individual’s 3/5-year overall survival. The SIPS was a key risk point in the nomogram (Additional file 1: Fig. S1I). The calibration curves and AUC curves of the Affy cohort, the TCGA cohort, and the Agilent cohort were presented in Additional file 1: Fig. S2A–L, respectively. The calibration curves fitted well to the ideal curve except for the testing part of the Affy cohort, the TCGA cohort, and especially the calibration curves of 5-year overall survival. However, AUCs of the nomogram model for predicting 3-year overall survival were 0.61, 0.58, and 0.57 in the Affy cohort, the TCGA cohort, and the Agilent cohort, respectively. Altogether, these findings indicated that the nomogram required improvement.

To further characterize the biological and clinical differences between the high and low SIPS subtypes, a TCGA cohort containing 379 OV patients was adopted for stratified analysis. Regarding clinical subtypes, patients at the high FIGO stage had higher SIPS levels than those at the low FIGO stage (Additional file 1: Fig. S2M). The debulking status was significantly correlated with the SIPS levels. Patients with optimal debulking status had lower SIPS levels than those with suboptimal status (Additional file 1: Fig. S2N). Furthermore, patients sensitive to platinum therapies had slightly lower SIPS values than those with platinum resistance (Additional file 1: Fig. S2O). Thus, the SIPS level could predict the clinical status of OV patients.

### The immune therapeutic benefit of the SIPS index

**Fig. 3:**
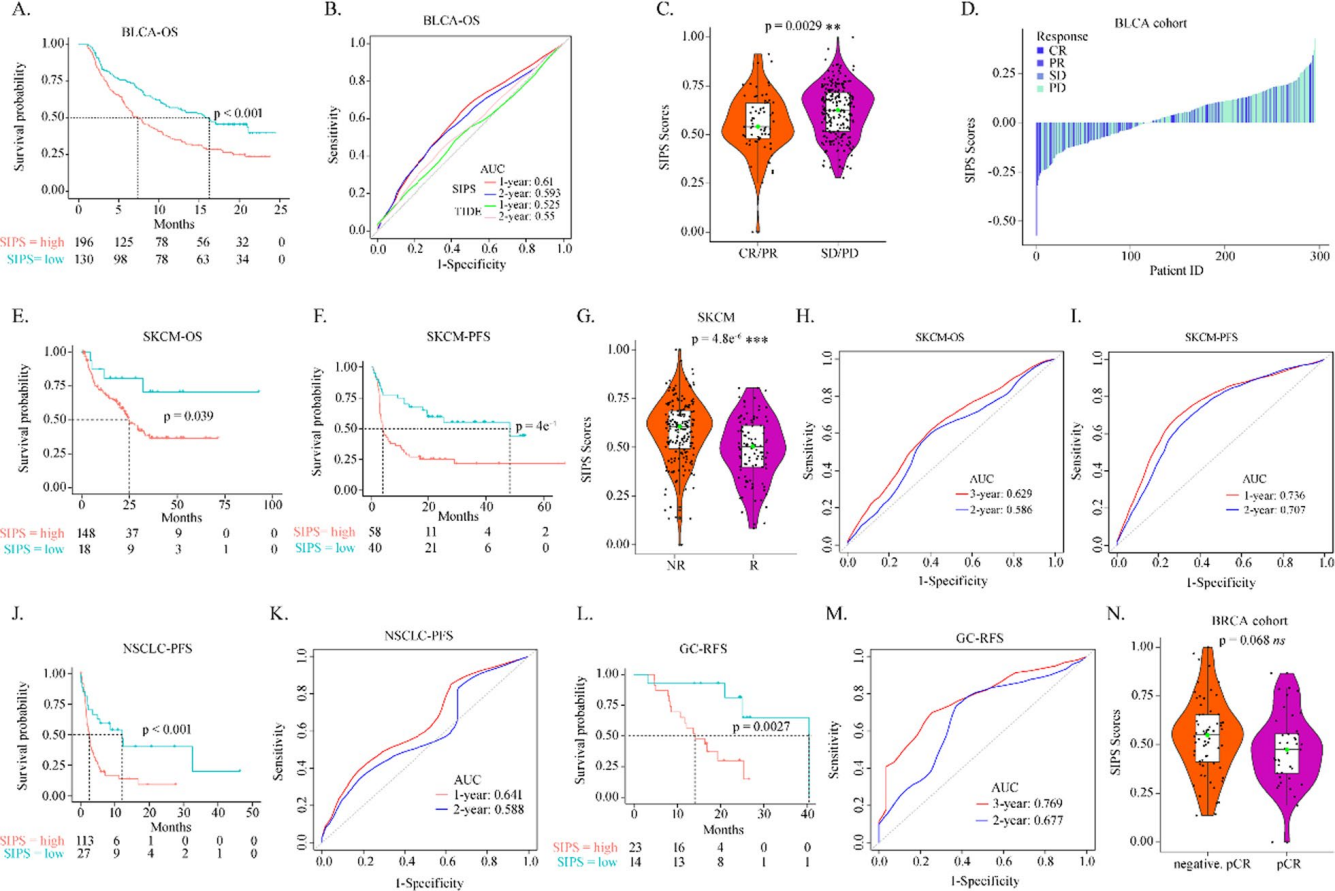
The immune therapeutic benefit of the SIPS index. **A** The Kaplan–Meier estimate of the overall survival in BLCA cohort, divided by two SIPS subtypes. **B** The time-dependent ROC curve at 1/2-year OS in BLCA cohort. **C** The boxplot showed the levels of SIPS score of patients with different immunotherapy responses in BLCA cohort. **D** The waterfall plot illustrated the distribution of SIPS in patients with different immunotherapy responses in BLCA cohort. The Kaplan–Meier estimate of the (**E**) overall survival and **F** progress free survival of SKCM cohort, divided by two SIPS subtypes. **G** The boxplot showed the levels of SIPS score of patients with different immunotherapy responses in SKCM cohort. **H** The time-dependent ROC curve at 1/2-year OS in SKCM cohort. **I** The time-dependent ROC curve at 1/2-year PFS in SKCM cohort. **J** The Kaplan–Meier estimate of the progress free survival of NSCLC cohort, divided by two SIPS subtypes. **K** The time-dependent ROC curve at 1/2-year PFS in NSCLC cohort. **L** The Kaplan–Meier estimate of the relapse-free survival of GC cohort, divided by two SIPS subtypes. **M** The time-dependent ROC curve at 1/2-year of RFS in the NSCLC cohort. **N** The boxplot showing the levels of SIPS score of patients with different immunotherapy responses in BRCA cohort

Due to the lack of published transcriptome of ovarian cancer patients who received immunotherapy, five integrated immunotherapy cohorts from the bladder urothelial carcinoma (BLCA), skin cutaneous melanoma (SKCM), non-small cell lung cancer (NSCLC), Gastrointestinal cancer (GC), and invasive breast carcinoma (BRCA) were selected to validate the predictive value of SIPS. Similar to before, patients were stratified into high and low SIPS subtypes by corresponding optimal cutoff values. In the BLCA cohort, patients in the high SIPS group had significantly worse overall survival than those with low SIPS (16.23 versus 7.39 months) (Fig. 3A). The time-dependent ROC analysis showed that the AUCs of the SIPS prognostic model for 1/2-year overall survival were 0.61 and 0.593, respectively, which were higher than that of TIDE predictive model (Fig. 3B) [22]. The SIPS values of BLCA patients with CR/PR were significantly lower than those with SD/PD (Fig. 3C). The predictive value of SIPS was also confirmed by the waterfall plots (Fig. 3D). In SKCM cohort, patients with low SIPS had a better prognosis than those with high SIPS (Fig. 3E, F). Furthermore, the AUCs of the SIPS prognostic model for 2/3-year overall survival were 0.586 and 0.629, respectively (Fig. 3G), and in the SKCM cohort, the AUCs for 1/2-year progress-free survival were 0.736 and 0.707, respectively (Fig. 3H). Immunotherapeutic responders had lower SIPS levels than the unresponsive ones (Fig. 3G). The predictive value of SIPS was also confirmed by the waterfall plots in the SKCM cohort (Additional file 1: Fig. S2P). Interestingly, SIPS value didn’t change significantly during immunotherapy (Additional file 1: Fig. S2Q).

Moreover, in the NSCLC cohort, SIPS value was also substantially anti-correlated with the progress-free survival (16.23 versus 7.39 months) (Fig. 3J). The AUCs for 1/2-year progress-free survival were 0.641 and 0.588, respectively (Fig. 3K). In the GC cohort, patients with low SIPS had substantially longer relapse-free survival than those with high SIPS (16.23 versus 7.39 months) (Fig. 3L), and the AUCs for 2/3-year relapse-free survival were 0.677 and 0.769, respectively (Fig. 3M). In the BRCA cohort, the patients with complete pathological responses had slightly higher SIPS than those without complete pathological responses (Fig. 3N). In conclusion, SIPS could predict the immunotherapeutic benefit among these cancers.

### The tumor microenvironmental landscape between the high and low SIPS patients

**Fig. 4:**
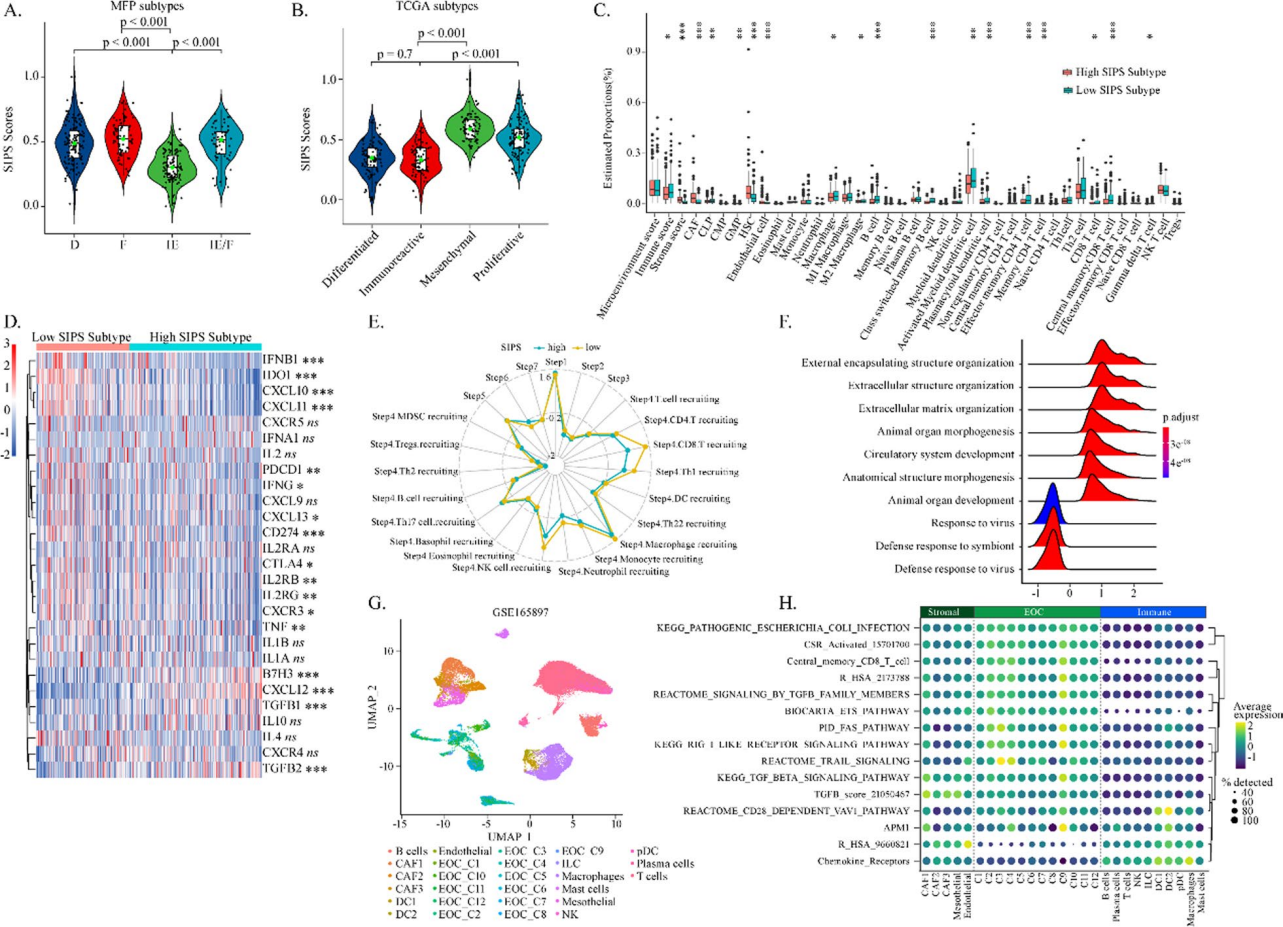
The tumor microenvironmental landscape between the high and low SIPS patients. The boxplot showed the levels of SIPS score of patients with different immune subtypes including **A** MFP and **B** TCGA subtypes in TCGA-OV cohort. **C** The boxplots showed the tumor microenvironmental components estimated by *xCell* algorithm in TCGA-OV cohort. **D** The heatmap showed the expression levels of several immune-related key genes in patients with different SIPS subtypes in Affy cohort. **E** The radar plot showed the correlation between SIPS and the tracking tumor immunophenotype in TCGA-OV cohort. **F** The ridge plot showed the enriched GO biological progress of the 150 hub genes derived from the WGCNA-STRING-Cytoscape analysis. **G** The UMAP plot showed a total of 51,786 cells in tumor microenvironment, and cell clusters were color-coded and labeled according to the original definition in the GSE165897 dataset. **H** The dot plot showed the levels of the 15 immune-related signatures derived from SIPS in the tumor microenvironment components

TME is intimately related to clinical immunotherapeutic response and can be classified into several subtypes. Our study indicated that the inflamed immune subtypes, including the immune-enriched/non-fibrotic (IE) subtype and immunoreactive subtype, had the lowest SIPS level compared to other TME subtypes (Fig. 4A, B). Differential immune landscapes between high and low SIPS subtypes are displayed in Fig. 4C. Patients with low SIPS had higher immune scores, while those with high SIPS had higher stroma scores. In detail, patients with low SIPS had higher proportions of cell types, including macrophages, class-switched memory B cells, activated myeloid dendritic cells, plasmacytoid dendritic cells, effector memory CD4^+^T cells, central memory CD8^+^T cells, and NKT cells. Patients with high SIPS had higher proportions of cancer-associated fibroblasts (CAFs), a major stromal component in TME. The Agilent cohort also found similar tumor environment compositions (Additional file 1: Fig. S3A).

Moreover, several immune-related key molecules were found to be significantly different between the two SIPS subtypes (Fig. 4D). The expression levels of several key cytokines, such as IFNB1, IFNG1, and TNF, were substantially higher in the low SIPS subtype. In contrast, the TGFB1 level was much higher in the high SIPS subtype. Although IL2 expressions were not significantly differentiated between the two SIPS subtypes, the IL2 receptor subtype IL2RA/B expression was markedly higher in the low SIPS subtype. Correlations between SIPS and several crucial immune checkpoint molecules, including PD-1, PD-L1, IDO1, CTLA-4, and B7H3, were investigated (Fig. 4D). Expression levels of PD-L1, IDO1, and CTLA-4 were more significant in low IRRS subtype than in high SIPS subtype. Meanwhile, B7H3 expression was much higher in the high SIPS subtype. Chemokines, including CXCL9/10/11/12/13, were key attractants that can promote immune cells infiltrating into TME. In this study, CXCL10/11, and their receptor CXCR3 were examined to be much more highly expressed in the low SIPS subtype than in the high SIPS subtype, while CXCL12 expression was much higher in the high SIPS subtype. Consistently, SIPS level was significantly associated with the infiltration of CD8^+^T cells and NK cells during the cancer-immunity cycle (Fig. 4E). The association between TME and SIPS was like that in the BLCA cohort (Additional file 1: Fig. S3B). The Exclusion, TIDE, and Dysfunction scores were substantially higher in the high SIPS subtype than in the low SIPS subtype (Additional file 1: Fig. S3B–C). Similarly, MDSCs and CAFs were significantly upregulated in the high SIPS subtype (Additional file 1: Fig. S3B, D, E).

The WGCNA algorithm was adopted to analyze the differentiated expressions of genes between the low and high SIPS subtypes to identify the different hub functional modules between the two subtypes (Additional file 1: Fig. S3F–I). Among five different co-expression modules, SIPS level was significantly anti-correlated with the yellow module and positively correlated with the other four modules (Additional file 1: Fig. S3I).

*STRING-Cytoscape* analysis was utilized to identify 150 hub genes from all the modules. The 150 hub genes were clustered into two significant signaling subnetworks (Additional file 1: Fig. S4A) and enriched in the immune-related and stroma-related subnetworks (Additional file 1: Fig. S4B). The immune-related subnetwork, including response to the virus, was significantly downregulated in the high SIPS subtype, while the stroma-related subnetwork, including extracellular structure organization, was significantly upregulated in the high SIPS subtype (Fig. 4F). Thus, stroma components were mainly enriched in the TME in high SIPS subtype, which could suppress immune cell infiltration and immune response. Furthermore, microenvironment cell types characterized by SIPS were analyzed based on a single-cell ovarian cancer dataset (Fig. 4G). In TME, a large part of SIPS-related signatures were consistently highly activated in the stromal components and tumor cells (Fig. 4H). We explored three datasets containing OV-associated stroma and epithelial tumor samples to determine the different activation of SIPS-related signatures between stroma cells and tumor cells (Additional file 1: Fig. S4C–E). Stroma components had higher activation of Chemokine receptors signaling and TGFB signaling than the tumor components. Conclusively, the stroma components in TME presented high SIPS levels and contributed to the formation of an immunosuppressive microenvironment.

### Correlation between SIPS and genomic instability

**Fig. 5:**
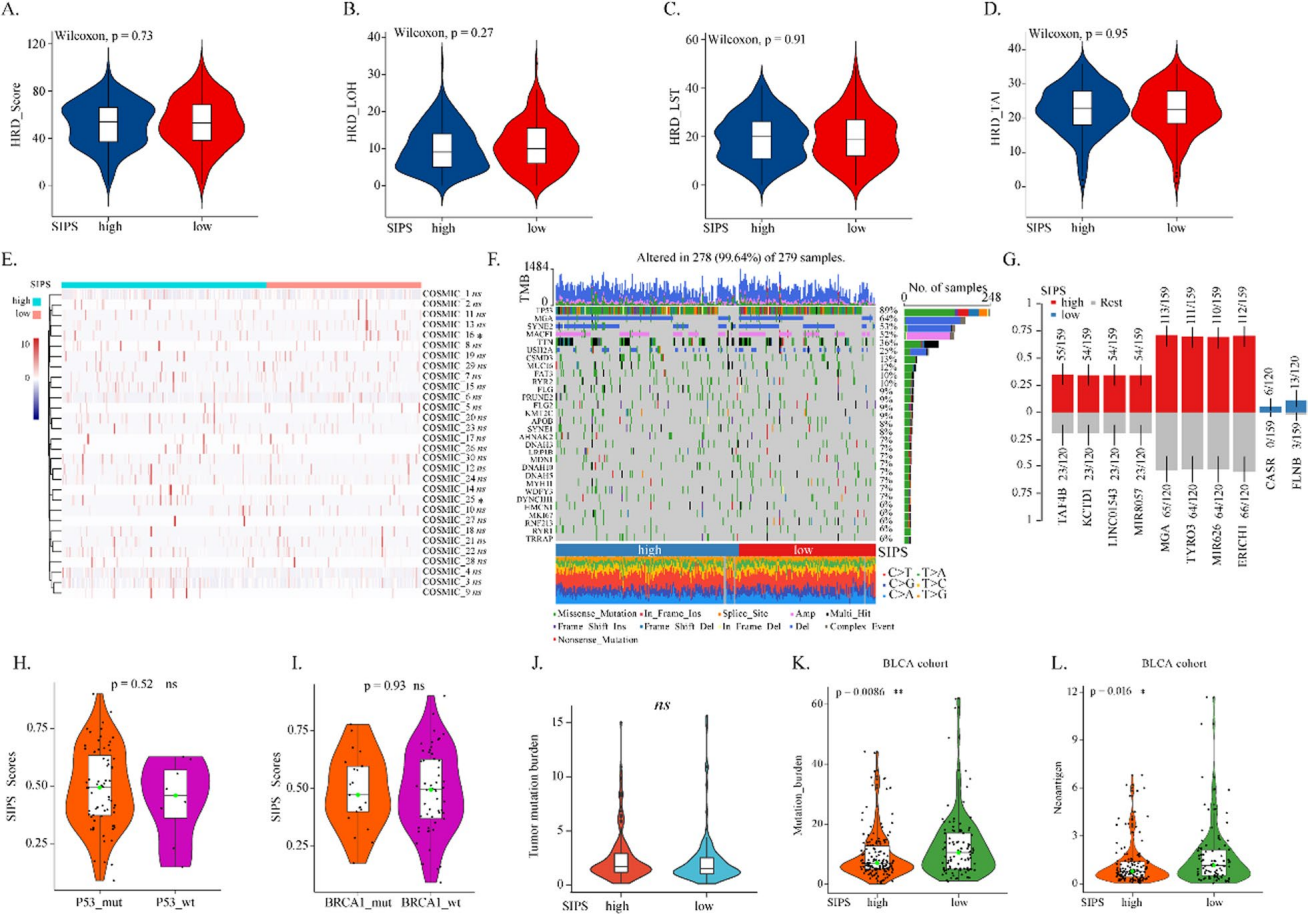
Correlation of SIPS with genomic instability. The boxplots showed the levels of **A** HRD Score, **B** LOH, **C** LST, and **D** TAI in patients with different SIPS subtypes in TCGA-OV cohort. **E** The heatmap showed the levels of the 30 most mutational signatures in patients with different SIPS subtypes. **F** The oncoprint showed the landscape genome changes in patients with different SIPS subtypes. **G** The bar plot showed the most mutated genes in patients with different SIPS subtypes. The boxplots showed the levels of SIPS score in patients with different **H** P53 and **I** BRCA1 mutational statuses in TCGA-OV cohort. **J** The boxplot showed the levels of tumor mutation burden in patients with different SIPS subtypes in TCGA-OV cohort. The boxplots showed the levels of **K** tumor mutation burden and **L** neoantigen in patients with different SIPS subtypes in BLCA cohort

Almost half of OV harbor homologous recombination deficiency (HRD) [23]. HRD causes tumor genomic instability, which induces considerable TMB and neoantigen load, activating the innate immune system [24]. There was no significant difference between SIPS subtypes and HRD scores, including LOH, LST, and TAI (Fig. 5A–D). Only two mutational signatures were found different between the low SIPS subtype. The high SIPS subtype, namely COSMIC 16/25, which were observed in liver cancer and Hodgkin lymphoma without specific etiology (Fig. 5E). Furthermore, few gene mutations were found different between the low SIPS subtype and the high SIPS subtype, except for TAF4B, KCTD1, and CASR, et al. (Fig. 5F, G). OV patients with P53 or BRCA1 mutation had similar SIPS levels compared to those with the wild-type (Fig. 5H, I). No significant TMB difference was found between the SIPS subtypes (Fig. 5J). However, in the BLCA cohort, patients with low SIPS had more mutation burden and neoantigens than those with high SIPS (Fig. 5K, L). In summary, SIPS was slightly associated with genomic instability in OV, indicating SIPS could not function on tumor antigenicity.

### Targeting to stroma promoted anti-tumor immunity

**Fig. 6:**
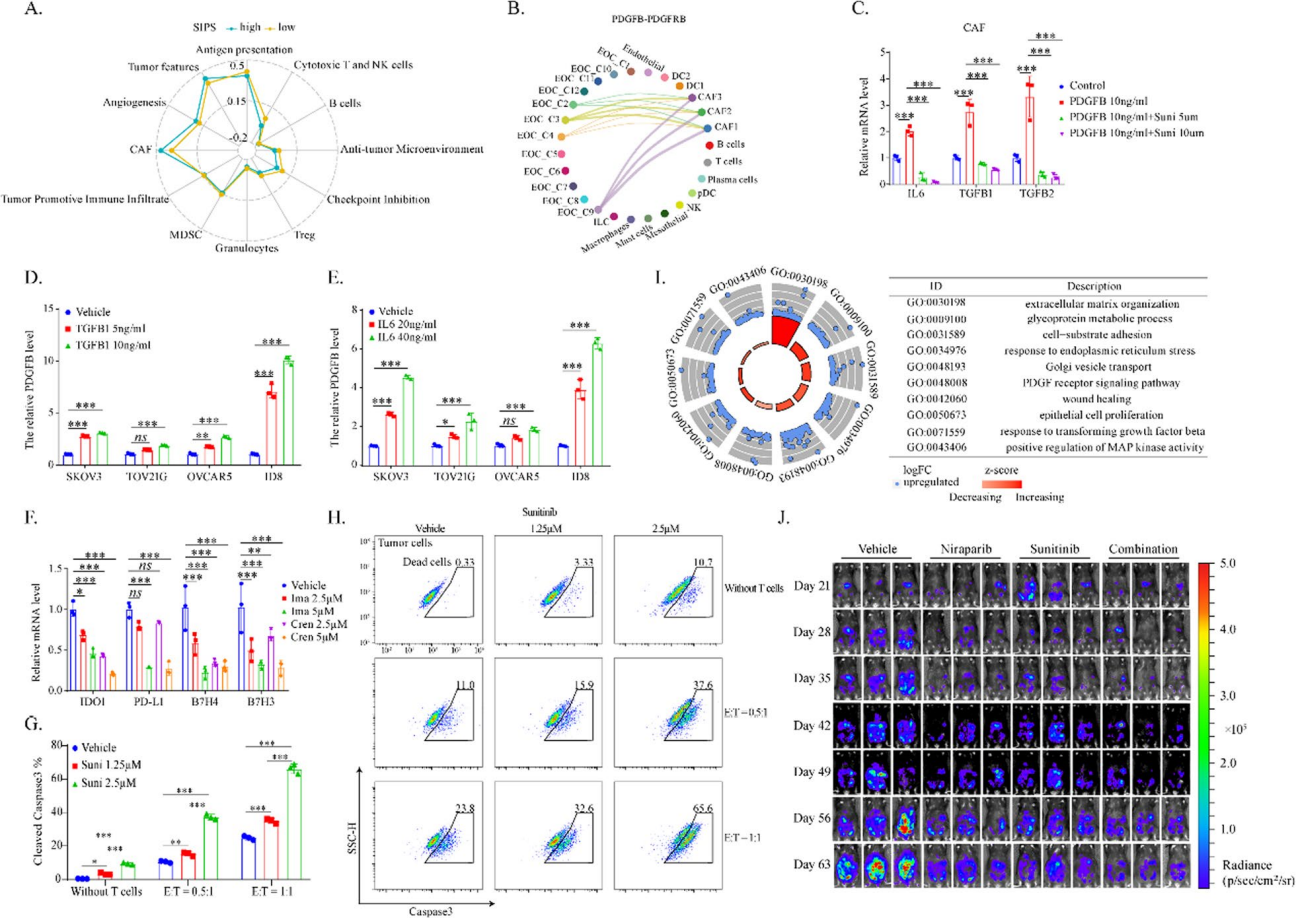
Targeting stroma promoted anti-tumor immunity. **A** The radar plot showed the correlation between SIPS and the immunogram developed by Bagaev et al. [12] to explore the cancer-immunity interactions in TCGA-OV cohort. **B** The dot plot showed the PDGFB–PDGFRB signaling among the tumor microenvironmental components in GSE165897 dataset. **C** The boxplot showed the expression levels of IL6 and TGFB1/2 in CAFs following 24 h-treatment of PDGFB and Sunitinib. **D** The boxplot showed the PDGFB expression levels in SKOV3, TOV21G, OVCAR5, and ID8 cells following 24 h-treatment of TGFB1. **E** The boxplot showed the PDGFB expression level in SKOV3, TOV21G, OVCAR5, and ID8 cells following 24 h-treatment of IL6. **F** The boxplot showed the expression levels of the immune checkpoint molecules IDO1, PD-L1, B7H4, and B7H3 in OVCAR5 cells following 24 h-treatment of the PDGFRB inhibitors Crenolanib and Imatinib. The **G** boxplot and **H** pseudo color plot showed the percentage of cleaved caspase-3 positive cells in Trp53^−/−^Brca2^−/−^-ID8 cells. The cells were pre-treated by PDGFRB inhibitor Sunitinib, followed by co-culturing with activated OT-1 T cells for 3 h. **I** The circle plot showed the enriched GO biological processes following Niraparib treatment in GSE164088 dataset. **J** Bioluminescence of C57BL/6j mice after inoculation of Trp53^−/−^Brca2^−/−^-ID8-luc cells

Abundant stroma indicated a worse prognosis for patients with high SIPS, based on which we hypothesized that stroma-targeting drugs could enhance the immunotherapeutic effect by reversing the suppressive immune microenvironment. As shown in Fig. 6A, the stroma of the SIPS subtype indeed presented more immunosuppressive components, including CAF and angiogenesis. The fibroblast marker PDGFRB was mainly expressed in cancer-associated fibroblasts in OV (data not shown) [25]. Moreover, OV cell-derived PDGFB was the primary source that activated PDGFRB in CAFs, indicating tumor cells promoted CAF formation (Fig. 6B). By checking the signaling network between CAFs and cancer cells, IL6 signaling was found to be of significance (Additional file 1: Fig. S5A). IL6 was a potent pro-tumor stimulator and strongly interacted with other hub genes in the SIPS network (Additional file 1: Fig. S4A). Hence, we proposed that immune-suppressive stroma could be vigorously promoted through a positive feedback loop of PDGFB signaling and IL6 signaling between cancer cells and CAFs. IL6 and TGFB1/2 expression was significantly upregulated by treating CAFs with PDGFB stimulation in vitro, which was blocked by PDGFRB inhibitors (Fig. 6C, Additional file 1: Fig. S5B). Accordingly, PDGFB expression in cancer cells was significantly upregulated by IL6 and TGFB1 in vitro (Fig. 6D, E). Previous studies indicated that PDGFRB-associated pathways were also activated in OV cells, which hindered survival [26]. Data in Fig. 6F, Additional file 1: Fig. S5C indicated that PDGFRB blockade inhibited the expression of immune checkpoint molecules in cancer cells, including IDO1, PD-L1, B7H3, B7H4, and Tim-3. OT-I killing assays verified that PDGFRB blockade enhanced tumor apoptosis by CD8^+^T cells (Fig. 6G, H, Additional file 1: Fig. S5D–E). Poly (ADP-ribose) polymerase inhibitors (PARPi), agents with specific efficacy in HRD ovarian cancer, activated the extracellular matrix-related pathways and PDGF receptor signaling in tumor stroma cells (Fig. 6I, Additional file 1: Fig. S5F). Moreover, the combination of PARPi Niraparib and PDGFB inhibitor significantly promoted IL6 expression of fibroblasts (Additional file 1: Fig. S5G). Activating stroma-related pathways and IL6 signaling could induce PARPi resistance in OV. Based on the results of in vitro experiments, the combination of PARP inhibitor and PDGFRB inhibitor was applied to treat OV in vivo. PDGFRB and VEGFR2 can be inhibited by Sunitinib with IC50s of 2 nM and 80 nM, respectively [27]. Hence, it was hypothesized that Sunitinib could reverse the immune-suppressive stroma by potently inhibiting CAFs and angiogenesis. Data in Fig. 6J, Additional file 1: Fig. S5H illustrated that Niraparib and Sunitinib inhibited Trp53^−/−^Brca2^−/−^-ID8-luc cancer cells growth in vivo. In summary, stroma-targeting PDGFRB and PARP inhibitors could significantly inhibit tumor growth.

### Construction of the convenient SIPS prognostic model in ovarian cancer

**Fig. 7:**
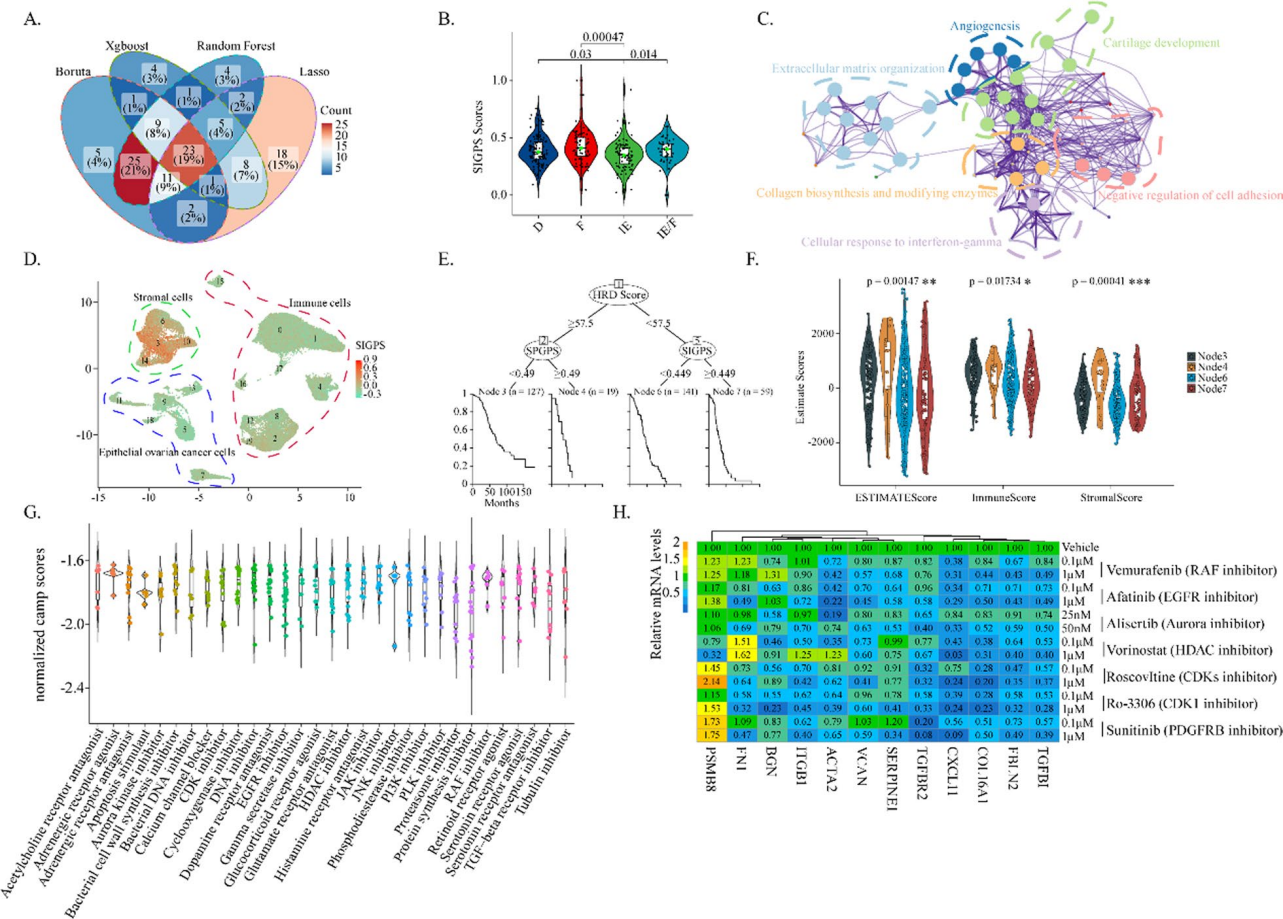
Construction of the convenient SIPS prognostic model in ovarian cancer. **A** The Venn diagram showed the common genes of the four machine-learning methods (*Boruta*, *Xgboost*, *Random Forest*, and *Lasso*). **B** The boxplot showed the levels of SIGPS in patient with different MFP subtypes in TCGA-OV dataset. **C** The network plot showed the enriched functional modules of SIGPS genes. **D** The UMAP plot showed the levels of the SIGPS in tumor microenvironment in GSE165897 dataset. The SIGPS level of each cell was calculated by the *AddModuleScore* function based on R package *Seurat*. **E** The decision tree of HRD and SIGPS in TCGA-OV cohort. In the tree, Node 3: HRD^+^SIGPS^−^; Node 4: HRD^+^SIGPS^+^; Node 6: HRD^−^SIGPS^−^; Node 7: HRD^−^SIGPS^+^. **F** The boxplot showed the estimate score levels of Estimate Score, Immune Score, and Stroma Score among the four subgroups of the decision tree in TCGA-OV dataset. **G** The boxplot demonstrated the normalized camp scores of agents suppressing the SIGPS, and the horizontal axis showed the agent types. Each dot in the plot represented one agent. **H** The heatmap showing the expression levels of stroma-related genes, including TGFBI, FBLN2, and COL16A1, in the CAFs after the treatment of several drugs as indicated

The SIPS index included 15 immune-related pathways, out of which four overlapped with TGFB signaling, causing redundancy and inconvenience in clinical practice. Hence, four machine-learning methods, including *Bortua*, *Xgboost*, *Random Forest*, and *Lasso* algorithms, were applied to rebuild a more convenient SIPS prognostic model and get the hub genes that could predict the immune subtypes (MFP subtypes) in OV. The average expression of the 23 common genes produced by four learning methods was defined as the stroma-immune gene prognostic signature (SIGPS) (Fig. 7A). Consistently, the immune-enriched/Non-Fibrotic (IE) subtype had the lowest SIGPS level among the conserved cancer microenvironment subtypes (Fig. 7B). The pathway enrichment analysis unveiled that SIGPS level was significantly correlated with pathways including Extracellular matrix organization, Angiogenesis, Cartilage development, Negative regulation of cell adhesion, Cellular response to interferon-gamma, and Collagen biosynthesis and modifying enzymes (Fig. 7C). Single-cell analysis demonstrated that stromal components especially CAFs had the highest SIGPS level among the three components in TME (Fig. 7D). The majority of 23 common genes were highly expressed in stromal component (Additional file 1: Fig. S6A), and further CAF clustering showed the highest SIGPS level in myofibroblast (Additional file 1: Fig. S6B–D). Together, like SIPS, SIGPS could reflect the immune suppressive stroma. SIGPS was further integrated with histologic grade, FIGO stage, and debulking status to predict overall survival in OV. The c-indices of the SIGPS prognostic model in indicating the overall survival were 0.63, 0.639, and 0.63 in the TCGA-OV cohort, Affy cohort, and Agilent cohort, respectively. The AUCs of the SIGPS prognostic model for 3/5-year overall survival were 0.603/0.568, 0.57/0.572, and 0.531/0.556 in the TCGA-OV cohort, Affy cohort, and Agilent cohort, respectively (Additional file 1: Fig. S6E–G). Taken together, the SIGPS prognostic model required improvement.

Considering HRD was slightly negatively associated with SIGPS (Additional file 1: Fig. S6H), the combined prognostic value of SIGPS and HRD was further validated. According to the decision tree of SIGPS and HRD, there were four subgroups termed HRD^+^SIGPS^+^, HRD^+^SIGPS^−^, HRD^−^SIGPS^+^, HRD^−^SIGPS^−^ (Fig. 7E). The SIGPS substantially affected the overall survival of HRD patients, and the HRD^+^SIGPS^−^ subgroup had the best overall survival among four subgroups (Fig. 7E, Additional file 1: Fig. S6I). Furthermore, HRD, SIGPS, and other clinical information were integrated to construct the nomogram. The SIGPS and HRD Score accounted for the most risk points compared to additional information (Additional file 1: Fig. S6J). The c-index of the nomogram model was 0.66 (0.62 ~ 0.70). Regarding TME, HRD patients had higher Immune Scores than HRP patients, and in the HRD subgroup, the SIGPS-positive patients had higher Stroma Scores (Fig. 7F). Taken together, the HRD ovarian patients with high SIGPS levels had much higher levels of immune suppressive stroma, which corresponded to a worse prognosis.

Drug-inducing expression signatures from the CMAP web were compared with SIGPS to screen SIGPS-suppressing drugs, which could enhance immunotherapeutic sensitivity and reverse the dire prognosis of HRD patients with high SIGPS levels. Remarkably, inhibitors that target the TGFB receptor, Aurora kinase, CDKs, HDACs, RAF signaling, EGFR signaling, etc., could significantly inhibit the SIGPS-like signature (Fig. 7G). Furthermore, the drug efficiency was examined with CAFs mainly expressing SIGPS signatures. As shown in Fig. 7H, drugs, including RAF inhibitor, EGFR inhibitor, Aurora inhibitor, HDAC inhibitor, CDK inhibitor, and PDGFRB inhibitor, significantly suppressed the expression of the many SIGPS-related genes. Thus, these agents had the potential to reverse poor survival of HRD OV patients with high SIGPS by inhibiting the immune suppressive stroma.

## Discussion

Although previous research suggested that ovarian cancer may be immunogenic, related clinical immunotherapy trials, haven’t achieved a promising outcome yet [5]. Developing a potent prognostic signature to identify patients sensitive to immunotherapy is urgent. In this study, the stroma-immune prognostic signature (SIPS) containing 15 immune-related pathways was constructed to predict the immunotherapeutic sensitivity of ovarian cancer. Patients with high SIPS had worse survival than their counterparts. Meanwhile, the responders in the immunotherapy cohorts had lower SIPS. Furthermore, the constructed nomogram indicated that SIPS was a critical prognostic factor among other clinical features such as grade. Higher SIPS levels were detected in patients at the advanced FIGO stage, in the suboptimal status, or from the platinum-resistant group, indicating that SIPS was significantly correlated with tumor progression. Therefore, SIPS could be a potent prognostic index for immunotherapy in ovarian cancer.

SIPS was significantly correlated with TME. Patients in the fibrotic, dessert, mesenchymal, or proliferative subtype tended to have high SIPS levels, while patients in the immune-enriched subtype had the lowest SIPS level. Notably, upregulated fibrotic components significantly reversed the SIPS level in the immune-enriched subtype. Patients with high SIPS levels had higher stroma scores and lower immune scores than those with low SIPS levels. The proportions of cancer-associated fibroblasts were significantly upregulated in the high SIPS subtype.

Meanwhile, the cytotoxic immune components, such as B cells, activated myeloid dendritic cells, and CD8^+^T cells, were significantly enriched in the low SIPS subtype. Furthermore, SIPS was anti-correlated with the expression levels of the IFNB1, IFNG, and the immune checkpoints molecules such as IDO1, PD1/PD-L1, and CTLA4, which indicated the potential applicative value of immune checkpoint blockades. Moreover, upregulated chemokines such as CXCL9/10/11/13 and related receptor CXCR3 in low SIPS subtype showed the recruiting step of CD8^+^T cells, Th1, and NK cells. Patients in the high SIPS subtype had a highly activated stroma and slightly activated immune modules. Almost 15 hub pathways in SIPS were highly expressed in stroma and tumor cells. However, SIPS was not dominantly induced tumor immunogenicity, considering that SIPS was only slightly correlated with genomic instability in OV. In conclusion, SIPS was significantly positively associated with stroma and anti-correlated with the immune, determining the positive correlation between SIPS level and immune suppression.

We found that the positive feedback loop of PDGFB signaling and IL6 signaling between cancer cells and CAFs could substantially benefit the immune-suppressive stroma. Inhibition of PDGFRB signaling could result in the down-regulation of IL6 and TGFB1/2 in CAFs and suppressed expression of immune checkpoints in tumor cells, leading to immune cytotoxicity. PDGFRB inhibitor Sunitinib can block CAFs activation and angiogenesis, and PARP inhibitor can promote stroma-related signatures, including PDGF receptor signaling. Theoretically, PGFRBi–PARPi combination could reverse the immune-suppressive stroma and further enhance tumor immunogenicity. The potential values of the combination were verified in vivo. SIGPS was further developed to identify immune subtypes based on the average expression of the 23 common genes to endow SIPS with better clinical practicability. Like SIPS, SIGPS was significantly positive-correlated with the stroma components. According to the decision model of SIGPS and HRD, high SIGPS corresponded to the substantially shortened overall survival of HRD patients. Several drug types that suppress the SIGPS-like signatures in CAFs were further identified, including RAF, EGFR, Aurora, HDAC, and CDK inhibitors, which could combine with immunotherapy.

Numerous studies have reported multiple immunological prognostic biomarkers in ovarian cancer [13]. Such biomarkers focused on tumor immunogenicity, such as the expression of cytokines and genomic instability in cancer cells. Other biomarkers directly estimated the proportions of immune cells in TME, including T cell activation and macrophage polarization. Additionally, most reported biomarkers were developed and tested with small sample size, and the immunotherapeutic prediction of many biomarkers was tested without immunotherapy cohorts. Furthermore, there needs to be more in vitro mechanical experiments and in vivo immunocompetent models to validate such biomarkers. In our study, the SIPS/SIGPS signature identified the key prognostic role of the immunosuppressive stroma components in ovarian cancer. The sample size was much larger than most studies before, which made it more robust. Although there were no available transcriptomic data of immunotherapy cohorts in ovarian cancer, the immunotherapeutic prediction of SIPS was validated by immunotherapy cohorts of several other epithelial cancers, such as melanoma and breast cancer. Indeed, the SIPS/SIGPS signature was further validated by various experiments, which indicated targeting the SIPS/SIGPS could substantially enhance immunotherapeutic efficacy in ovarian cancer.

The current study included several limitations as well. Firstly, the extraordinary intratumor or interpatient heterogeneity of ovarian cancer was not considered. Secondly, a large part of the collected cohorts in our study needed complete clinicopathological information, which made it impossible to identify whether the SIPS/SIGPS was an independent prognostic factor when the clinicopathological information was thoroughly adjusted. Thirdly, the c-indices and AUCs of SIPS or SIGPS were less than 0.7 in ovarian cohorts, but the AUCs of SIPS were higher in the immunotherapy cohorts. Despite the drawbacks, according to the extensive collection of ovarian cancer and immunotherapy cohorts, SIPS, as a predictive marker based on tumor microenvironment, is outstanding for its precision and efficiency. In conclusion, the SIPS model proved reliable for ovarian cancer survival prediction and therapy guidance.

## Conclusions

In summary, our findings clarified the SIPS/SIGPS as the critical negative indicator for the prognosis in ovarian cancer patients, which revealed the vital immunosuppressive role of microenvironmental tumor stroma. The combination of PARP inhibitors and agents targeting the SIPS/SIGPS-like signature in the stroma could substantially inhibit tumor growth, providing a promising immunotherapeutic strategy for treating HRD ovarian cancer.

### Supplementary Information

**Additional file 1: Figure S1.** Immune related signatures for the prognostic prediction of ovarian cancer. **Figure S2. **The immune therapeutic benefit of the SIPS index. **Figure S3. **The tumor microenvironmental landscape between the high and low SIPS patients. **Figure S4. **The tumor microenvironmental landscape between the high and low SIPS patients. **Figure S5. **Targeting stroma promoted anti-tumor immunity. **Figure S6. **Construction of the convenient SIPS prognostic model in ovarian cancer. **Figure S7. **The summarized presentation of the stroma-immune prognostic signature.

**Additional file 2: Table S1.** Dataset information.

**Additional file 3: Table S2.** 470 immune related signatures.

**Additional file 4: Table S3.** The Unicox results in the affy cohort.

**Additional file 5: Table S4. **Hub functional analysis.

**Additional file 6: Table S5.** cmap drug.

**Additional file 7: Table S6.** qPCR primers.

**Additional file 8: Table S7.** Cytokines inhibitors.

## Data Availability

All the datasets could be downloaded directly from the indicated websites. Datasets and custom scripts are available upon request.

## Abbreviations

SIPS: Stroma-immune prognostic signature
SIGPS: Stroma-immune gene prognostic signature
CAF: Cancer associated fibroblast
HRD: Homologous recombination deficiency
TIDE: Tumor Immune Dysfunction and Exclusion
CAMP: Connectivity Map
CR: Clinical remission
PR: Pathological remission
SD: Stable disease
PD: Progressive disease
