# Supplementary material for: Suppressive stroma-immune prognostic signature impedes immunotherapy in ovarian cancer and can be reversed by PDGFRB inhibitors
Source: J Transl Med. 2023 Sep 1;21:586. doi: 10.1186/s12967-023-04422-x (PMC10472577; doi:10.1186/s12967-023-04422-x)
Supplement: Supplementary file 1 — Additional file 1: Figure S1. Immune related signatures for the prognostic prediction of ovarian cancer. Figure S2. The immune therapeutic benefit of the SIPS index. Figure S3. The tumor microenvironmental landscape between the high and low SIPS patients. Figure S4. The tumor microenvironmental landscape between the high and low SIPS patients. Figure S5. Targeting stroma promoted anti-tumor immunity. Figure S6. Construction of the convenient SIPS prognostic model in ovarian cancer. Figure S7. The summarized presentation of the stroma-immune prognostic signature. [file 12967_2023_4422_MOESM1_ESM.docx]

**Additional Data**

| **Figure S1.** Immune related signatures for the prognostic prediction of ovarian cancer. |
| --- |
| **Figure S2.** The immune therapeutic benefit of the SIPS index. |
| **Figure S3.**The tumor microenvironmental landscape between the high and low SIPS patients. |
| **Figure S4.** The tumor microenvironmental landscape between the high and low SIPS patients. |
| **Figure S5.** Targeting stroma promoted anti-tumor immunity. |
| **Figure S6.** Construction of the convenient SIPS prognostic model in ovarian cancer. |
| **Figure S7.** The summarized presentation of the stroma-immune prognostic signature. |

**Additional figures:**


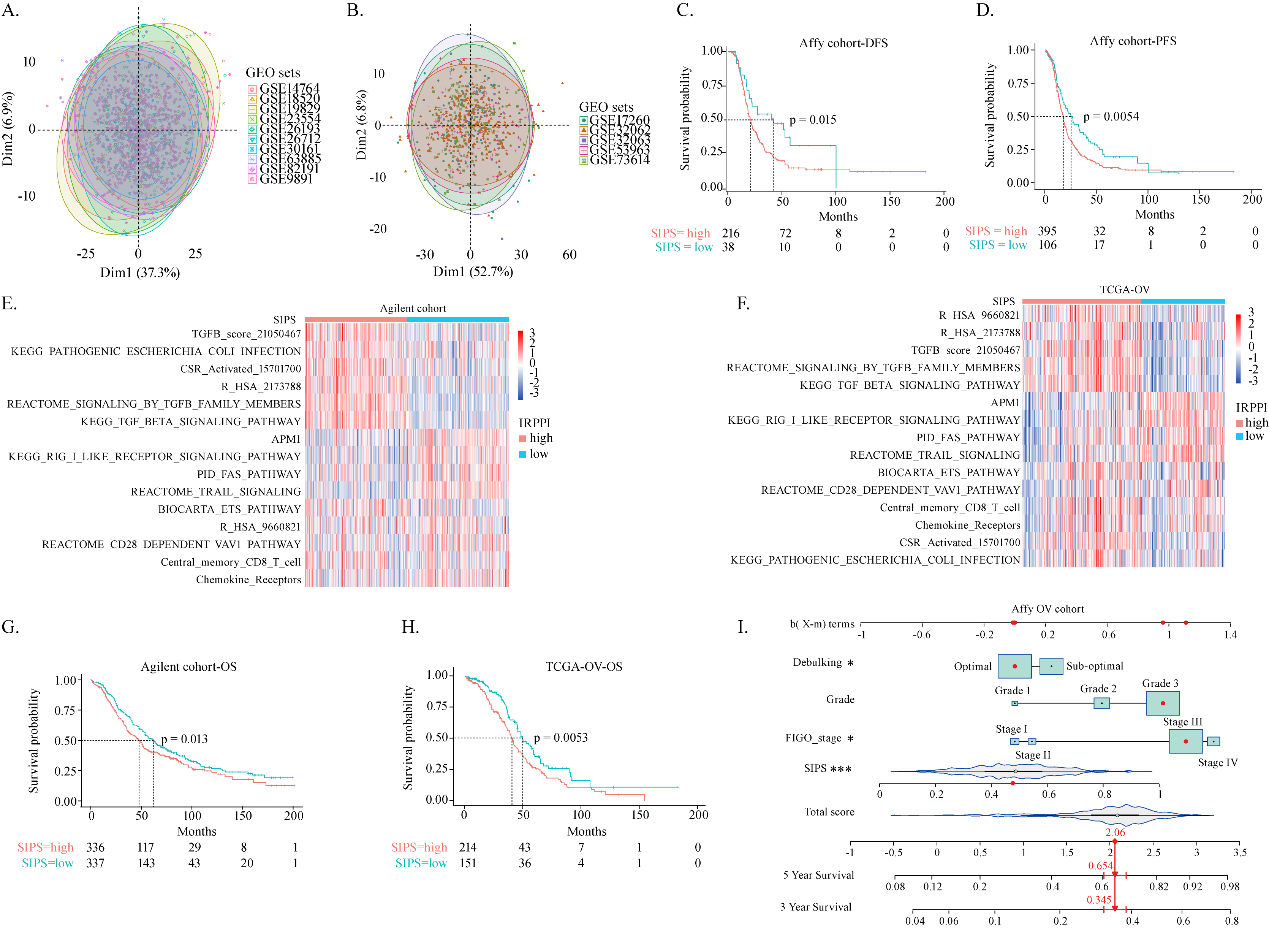


**Figure S1. Immune related signatures for the prognostic prediction of ovarian cancer**. The PCA plot showed the mitigation of the batch effect by combat algorithm in the (A) Affy cohort and (B) Agilent cohort. The Kaplan-Meier estimate of the (C) Disease free survival and (D) progress free survival of the Affy cohort, divided by the two SIPS subtypes. (E) The heatmap of Agilent cohort by using the NESs of 15 immune-related signatures. (F) The heatmap of TCGA-OV cohort by using the NESs of 15 immune-related signatures. The Kaplan-Meier estimate of the overall survival of the (G) Agilent cohort and (H) TCGA-OV cohort, divided by the two SIPS subtypes. (I) The nomogram developed for predicting the probability of 3/5-year overall survival in the Affy cohort. The red vertical arrow showed the survival probability of one individual.


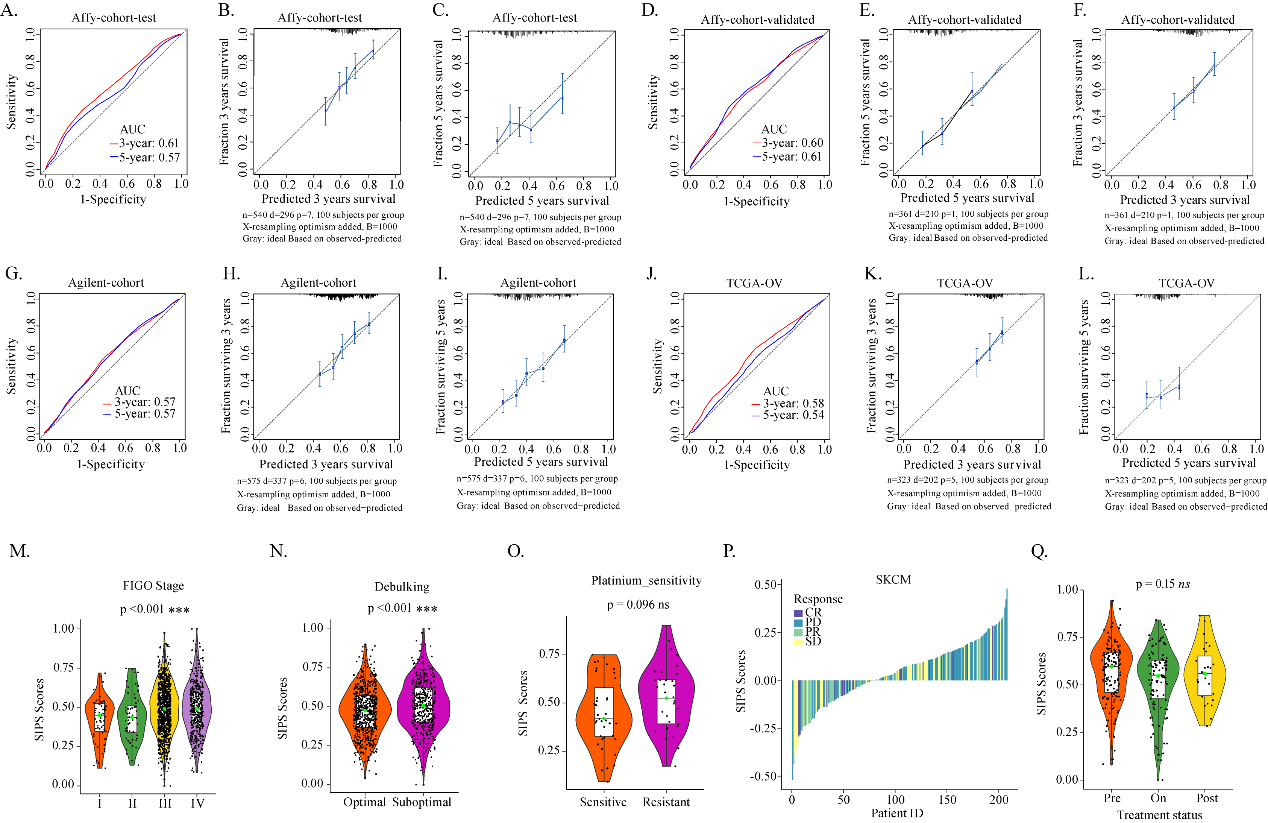


**Figure S2. The immune therapeutic benefit of the SIPS index**. (A) The time-dependent ROC curve at 3/5-year OS of the nomogram in the test subset of Affy cohort. Calibration plot of the nomogram in terms of agreement between the predicted and observed (B) 3-year and (C) 5-year outcomes in test subset of Affy cohort. The dashed lines along the 45-degree line show a nomogram's ideal performance. (D) The time-dependent ROC curve at 3/5-year OS of the nomogram in the validated subset of Affy cohort. Calibration plot of the nomogram in terms of agreement between the predicted and observed (E) 3-year and (F) 5-year outcomes in the validated subset of Affy cohort. (G) The time-dependent ROC curve at 3/5-year OS of the nomogram in the TCGA-OV cohort. Calibration plot of the nomogram in terms of agreement between the predicted and observed (H) 3-year and (I) 5-year outcomes in the TCGA-OV cohort. (J) The time-dependent ROC curve at 3/5-year OS of the nomogram in the Agilent cohort. Calibration plot of the nomogram in terms of agreement between the predicted and observed (K) 3-year and (L) 5-year outcomes in the Affy cohort. The boxplot showing the levels of SIPS score in patients with different (M) FIGO stages, (N) debulking statuses, and (O) platinum sensitivities in the Affy cohort. (P) The waterfall plot illustrating the distribution of SIPS for patients with different immunotherapy responses in the SKCM cohort. (Q) The boxplot showing the levels of SIPS score in patients with different treatment statuses in the SKCM cohort.


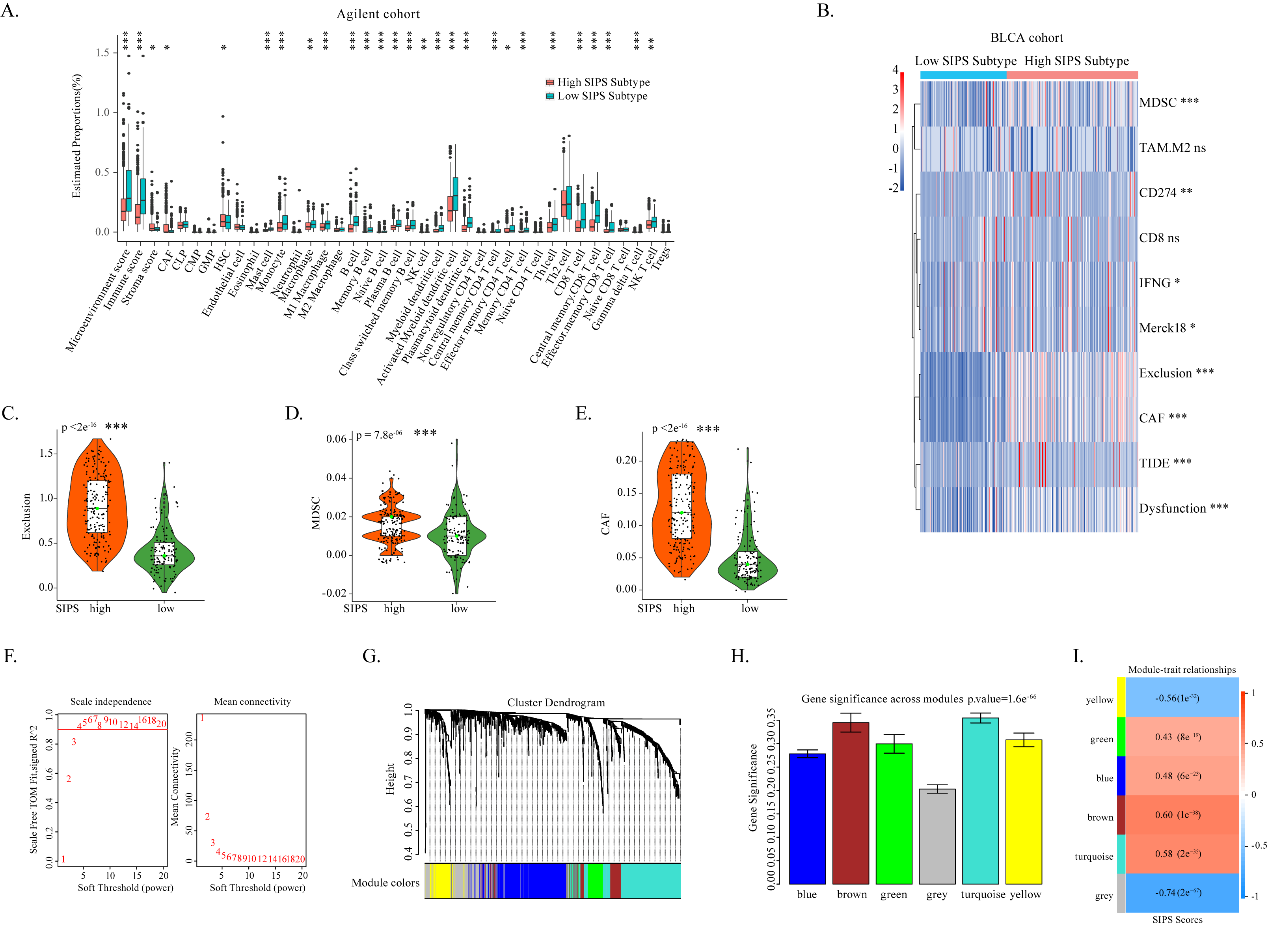


**Figure S3. The tumor microenvironmental landscape between the high and low SIPS patients**. (A) The boxplots showed the tumor microenvironmental components estimated by xCell algorithm in Agilent cohort. (B) The heatmap showing the levels of several immune-related components in patients with different SIPS subtypes in the BLCA cohort. The boxplots showing the levels of (C) Exclusion score, (D) MDSC, and (E) CAF in patients with different SIPS subtypes in the BLCA cohort. (F) Selection of the soft threshold with scale independence and mean connectivity in the TCGA-OV cohort. (G) Cluster dendrogram of the six modules. Genes in the gray module did not fit into any module and their expression patterns are not significantly connected. (H) Distribution of average gene significance and errors in the modules associated with the SIPS. (I) Correlation between the hub modules and the SIPS. The correlation between the hub modules and SIPS is shown by the correlation coefficient in each cell.


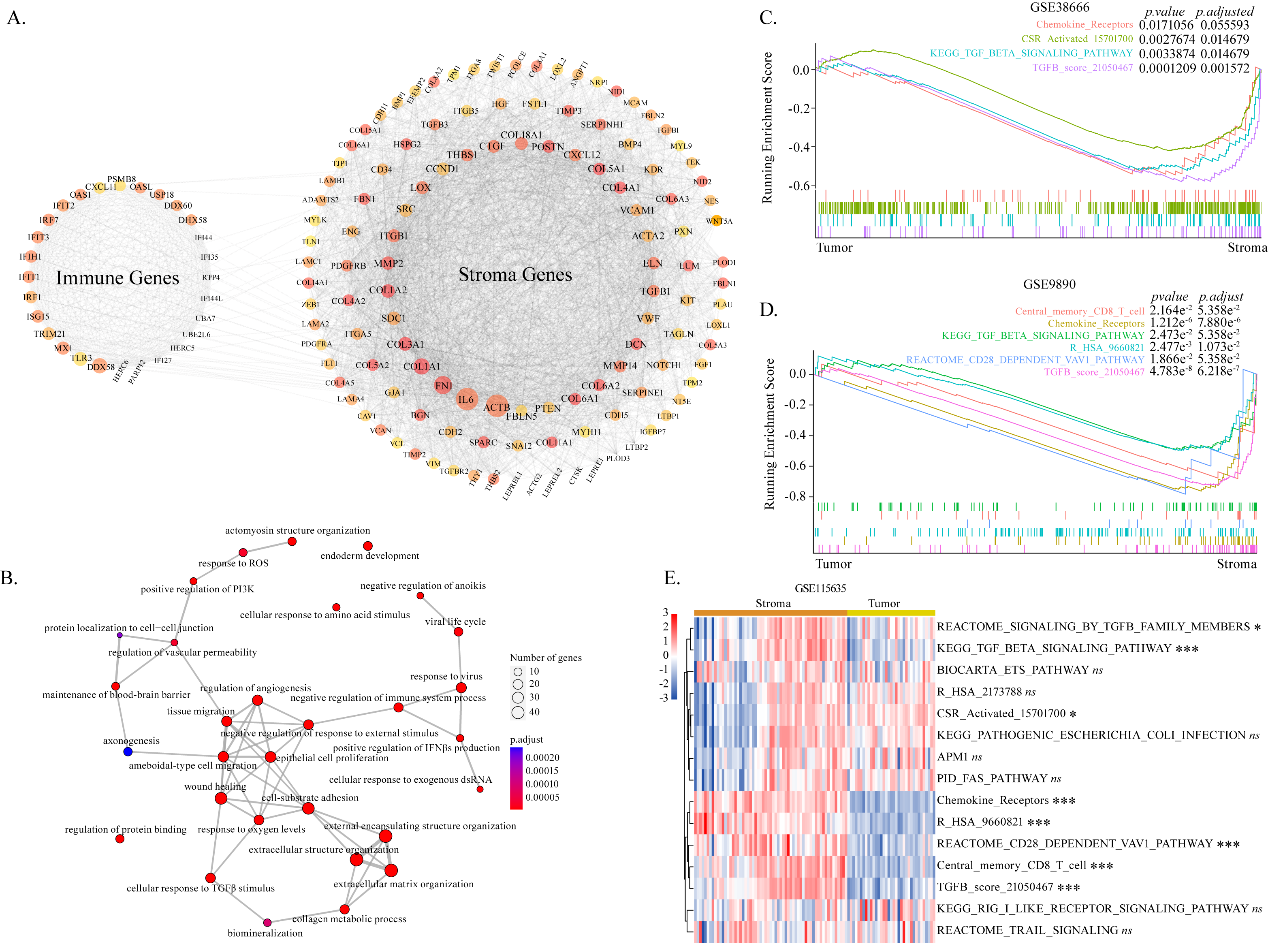


**Figure S4. The tumor microenvironmental landscape between the high and low SIPS patients**. (A) The correlation network of 150 hub genes generated by WGCNA-STRING-Cytoscape analysis. According to the CytoHubba's maximal clique centrality algorithm, the correlation degree grew as the circle's color changed from red to yellow. The diameter of the circle produced by the CytoHubba's betweenness algorithm could also serve as a proxy for the degree of connection. (B) The enrichment map showing the enriched GO biological processes of the 150 hub genes generated by WGCNA-STRING-Cytoscape analysis. The GSEA enrichment plot showing the enriched signatures of the SIPS in the stroma component from (C) GSE38666 and (D) GSE9890. (E) The heatmap showing the levels of the 15 signatures derived from the SIPS in different tissue compartments in the GSE115635 dataset.


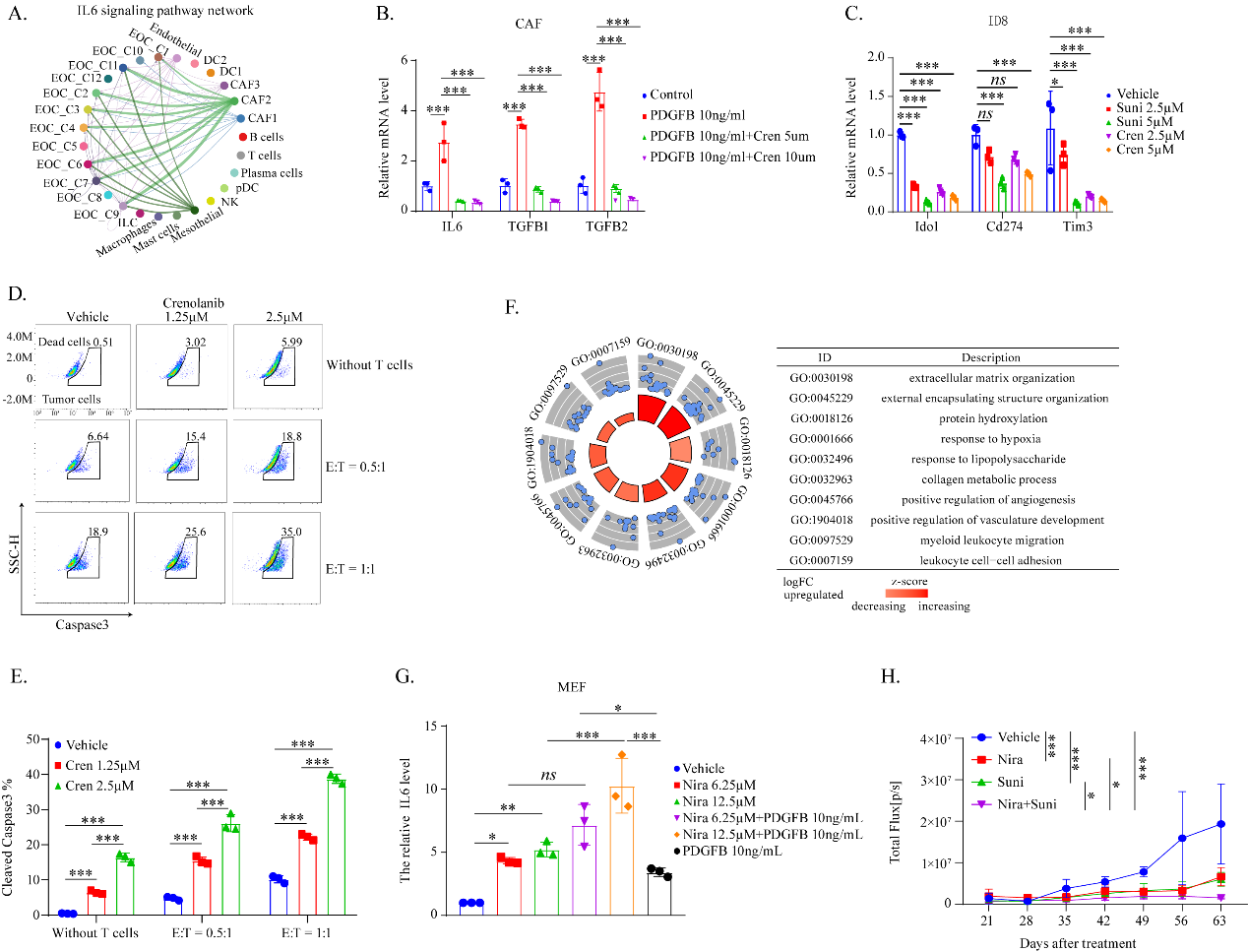


**Figure S5. Targeting stroma promoted anti-tumor immunity**. (A) The dot plot showing the IL6 signaling among the tumor microenvironmental components in the GSE165897 dataset. (B) The boxplot showing the expression levels of IL6 and TGFB1/2 in CAFs following the 24h-treatment of PDGFB and Crenolanib. (C) The boxplot showing the expression levels of the immune checkpoint molecules Ido1, Cd274, and Tim3 in ID8 cells following the 24h-treatment of the PDGFRB inhibitors Crenolanib and Sunitinib. The (D) pseudo color plot and (E) boxplot showing the percentage of cleaved caspase-3 positive cells in Trp53^-/-^Brca2^-/-^-ID8 cells. The cells were pre-treated by the PDGFRB inhibitor Crenolanib, followed by co-culturing with activated OT-1 T cells for 3 hours. (F) The circle plot showing the enriched GO biological processes following the Olaparib treatment in the GSE164088 dataset. (G) The boxplot showing the IL6 expression levels in CAFs following the 24h-treatment of PDGFB and Niraparib. (H) Tumor burden was evaluated by quantifying total flux with PE Living Image software. Data were presented by mean value of 5 mice per group ± standard error.


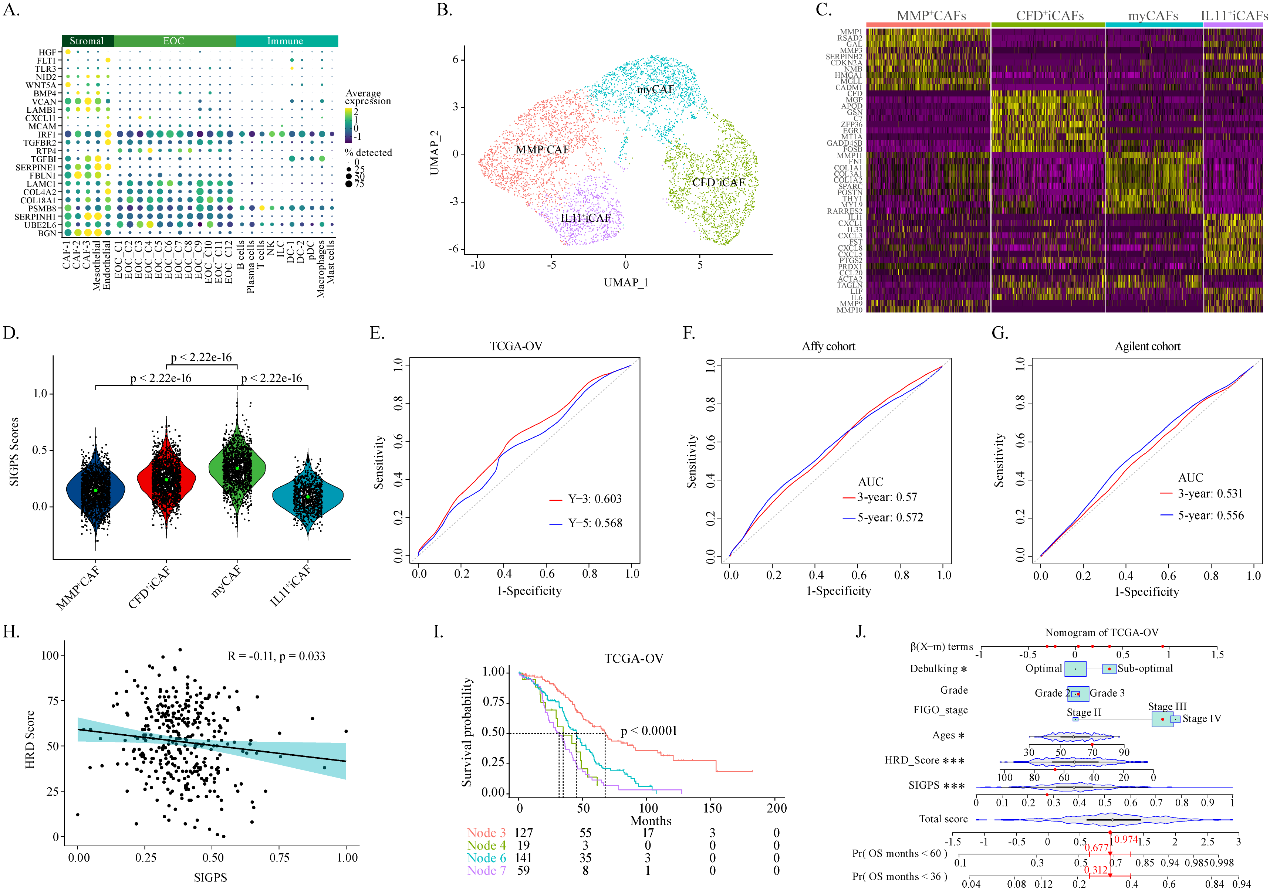


**Figure S6. Construction of the convenient SIPS prognostic model in ovarian cancer**. (A) The dot plot showing the levels of the 23 genes derived from the SIGPS in the tumor microenvironment components in the GSE165897 dataset. (B) The UMAP plot showed the CAF clusters in GSE165897 dataset. iCAF: inflammatory CAF; myCAF: myofibroblast. (C) The heatmap showed the significant different genes in each CAF cluster. (D) The boxplot showed the levels of SIGPS in the different CAF clusters in GSE165897 dataset. The SIGPS level of each cell was calculated by the *AddModuleScore* function based on R package *Seurat*. The time-dependent ROC curve at 3-year and 5-year OS of the nomogram in the (E) TCGA-OV cohort, (F) Affy cohort, and (G) Agilent cohort. (H) The scatter plotted for the correlation between HRD Score and SIGPS. (I) The overall survival in the four subgroups of the decision tree of HRD and SIGPS in the TCGA-OV cohort. (J) The nomogram developed for predicting the probability of 3- and 5-year overall survival in the TCGA-OV cohort. The red vertical arrow showed the survival probability of one individual.


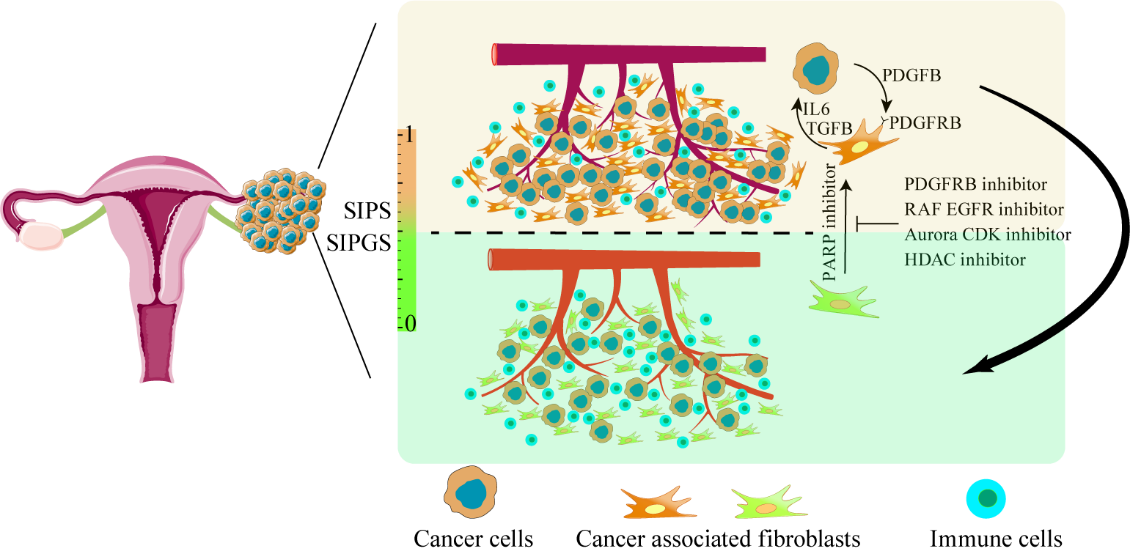


**Figure S7. The summarized presentation of the stroma-immune prognostic signature**. The summarized presentation of the stroma-immune prognostic signature. In the tumor microenvironment of ovarian cancer, the high SIPS/SIGPS level indicated the abundant immunosuppressive stroma, including the CAFs and angiogenesis. The immunosuppressive CAFs could secret IL6 and TGFB, activating the cancer cells, while cancer cells could secret PDGFB i­n the positive-feedback loop to further activate CAFs. The ­PARP inhibitors could promote the immunosuppressive CAFs. Several drug types, including PDGFRB, RAF, EGFR, Aurora, CDK, and HDAC inhibitors, might reverse the immunosuppressive CAFs to enhance immunotherapy efficacy.
